# Simulating the impact of greenspace exposure on metabolic biomarkers in a diverse population living in San Diego, California: A g-computation application

**DOI:** 10.1097/EE9.0000000000000326

**Published:** 2024-08-07

**Authors:** Anaïs Teyton, Nivedita Nukavarapu, Noémie Letellier, Dorothy D. Sears, Jiue-An Yang, Marta M. Jankowska, Tarik Benmarhnia

**Affiliations:** a Herbert Wertheim School of Public Health and Human Longevity Science, University of California, San Diego, California; b School of Public Health, San Diego State University, San Diego, California; c Scripps Institution of Oceanography, University of California, San Diego, La Jolla, California; d Population Sciences, Beckman Research Institute, City of Hope, Duarte, California; e Irset Institut de Recherche en Santé, Environnement et Travail, UMR-S 1085, Inserm, University of Rennes, EHESP, Rennes, France; f College of Health Solutions, Arizona State University, Phoenix, Arizona; g Department of Medicine, University of California, San Diego, La Jolla, California; h Department of Family Medicine, University of California, San Diego, La Jolla, California; i Moores Cancer Center, University of California, San Diego, La Jolla, California

**Keywords:** G-formula, Causal inference, Greenspace, Normalized difference vegetation index, Social determinants of health, Intervention simulation, Cardiometabolic disease, Metabolic syndrome

## Abstract

**Introduction:**

Growing evidence exists that greenspace exposure can reduce metabolic syndrome risk, a growing public health concern with well-documented inequities across population subgroups. We capitalize on the use of g-computation to simulate the influence of multiple possible interventions on residential greenspace on nine metabolic biomarkers and metabolic syndrome in adults (N = 555) from the 2014–2017 Community of Mine Study living in San Diego County, California.

**Methods:**

Normalized difference vegetation index (NDVI) exposure from 2017 was averaged across a 400-m buffer around the participants’ residential addresses. Participants’ fasting plasma glucose, total cholesterol, high-density lipoprotein cholesterol, low-density lipoprotein cholesterol, and triglyceride concentrations, systolic and diastolic blood pressure, hemoglobin A1c (%), waist circumference, and metabolic syndrome were assessed as outcomes of interest. Using parametric g-computation, we calculated risk differences for participants being exposed to each decile of the participant NDVI distribution compared to minimum NDVI. Differential health impacts from NDVI exposure by sex, ethnicity, income, and age were examined.

**Results:**

We found that a hypothetical increase in NDVI exposure led to a decrease in hemoglobin A1c (%), glucose, and high-density lipoprotein cholesterol concentrations, an increase in fasting total cholesterol, low-density lipoprotein cholesterol, and triglyceride concentrations, and minimal changes to systolic and diastolic blood pressure, waist circumference, and metabolic syndrome. The impact of NDVI changes was greater in women, Hispanic individuals, and those under 65 years old.

**Conclusions:**

G-computation helps to simulate the potential health benefits of differential NDVI exposure and identifies which subpopulations can benefit most from targeted interventions aimed at minimizing health disparities.

**What this study adds:**

This study improves understanding of the relationship between greenspace exposure and metabolic risk biomarkers and builds evidence for the potential positive effects of increasing green spaces in urban environments. As metabolic syndrome is a growing public health concern in the United States, identifying population-level strategies to reduce this risk is critical. G-computation, a flexible causal inference method, can be utilized to simulate potential targeted interventions on NDVI exposure and observe its possible health benefits. Additionally, effect modification can be assessed with g-computation to identify which subpopulations can benefit most from greening strategies. This has important implications on policy and interventions aimed at reducing the health burden of metabolic syndrome and promoting health equity.

## Introduction

Metabolic syndrome is a prominent public health issue in the United States that affects one in three adults, and it is a growing concern as metabolic health has worsened over time.^1–3^ Defining characteristics of metabolic syndrome include high blood pressure, abdominal obesity, high triglyceride levels, low high-density lipoprotein (HDL) cholesterol, and impaired fasting glucose.^2,4,5^ It is well-established that metabolic syndrome increases the risk of cardiovascular disease, including coronary heart disease, carotid artery disease, and ischemic stroke, diabetes, and premature cardiovascular-specific and all-cause mortality.^1,6^ Given these severe consequences to health, it is critical to identify who is most at risk and to prioritize reducing this risk, especially as metabolic syndrome is largely preventable.

Certain individuals are at greater risk of metabolic syndrome. For example, older adults (compared to younger adults), Hispanic and non-Hispanic Black individuals (compared to non-Hispanic White individuals), men (compared to women), and those with lower socioeconomic status (compared to those with higher socioeconomic status) have been shown to be at increased risk of metabolic syndrome.^7–9^ The prevalence of metabolic syndrome has increased over time, especially among these subgroups, leading to an increase in avoidable health inequalities.^10^ Thus, it is particularly important to focus on populations with elevated risk and identify potential interventions that may reduce these health inequities.

Assessing the influence of modifiable factors on metabolic syndrome is important to reduce the risk of metabolic syndrome and its health consequences. Certain risk factors for metabolic syndrome have been identified, including environmental exposures such as noise and air pollution.^11,12^ A few studies have also assessed the role of greenspace exposure, often measured as normalized difference vegetation index (NDVI), on metabolic syndrome biomarkers, where a protective relationship has been identified.^13–18^ Yet, this literature has mostly focused on estimating a traditional dose–response and has not provided actionable evidence based on interventions that simulate the influence of modifying NDVI exposure on metabolic biomarkers. Simulating interventions on greenspace can be implemented to understand the potential implications regarding changes in health outcomes resulting from changes in residential greenspace.

One approach to simulate the health impacts of potential interventions on NDVI exposure is g-computation. G-computation is an estimation technique used to calculate the average causal effect through standardization.^19,20^ Though a less frequently used causal inference method, g-computation allows for the effects of an intervention to be evaluated by estimating potential outcomes contrasts.^21,22^ It can estimate outcomes under a range of different exposure scenarios and be used to identify heterogeneity in the effects of these exposure scenarios in subgroups.^21,23^ This can help identify salient policies and interventions as the health benefits of multiple scenarios are quantifiable, and subpopulations that stand the most to gain from such interventions can be prioritized.

In this study, capitalizing on the application of g-computation, we simulated the etiological impact of differential greenspace exposure on nine metabolic biomarkers and metabolic syndrome in a cohort of adults living in San Diego County, California. We also assessed the effect modification of modeled relationships by several demographic characteristics to identify which subgroups would benefit most from increasing exposure to greenspace around their home environments.

## Methods

### Study population

The cross-sectional Community of Mine study aimed to assess the influence of environmental exposures and lifestyle behaviors on cancer risk in an ethnically diverse population living in San Diego County from 2014 to 2017. Participants were randomly selected from 764 census block groups from the 1794 total census block groups in San Diego County that varied with regard to the highest and lowest terciles of walkability and food access. The study ensured that no more than 10 participants were recruited from any given census block group. To be eligible for this study, participants had to be living for a minimum of 6 months in their current residence, be able to move without human assistance, travel to a study visit, have a phone, read and write fluently in either English or Spanish, provide informed consent, comply with the protocol, and complete all assessments. A total of 602 individuals aged 35–80 years were included in the study. Participants were scheduled for a clinical visit when the eligibility criteria were confirmed, and consent was received. During this clinical visit, blood pressure and a 12-hour fasted blood draw were collected. Additional measurements, such as waist circumference, were taken. At the visit, participants completed a self-report survey providing their demographic information. Further details on the study protocol and design can be found elsewhere.^24^ This study was approved by the University of California, San Diego Institutional Review Board (protocol #140510), and all participants provided signed informed consent. In this study, a complete case analysis was utilized, where those with missing information related to the NDVI exposure, metabolic biomarker outcomes, and confounders were removed (see Table S1; http://links.lww.com/EE/A293 regarding descriptive statistics on missingness), for a final sample size of 555 participants (92.2% retention).

### Greenspace exposure and metabolic biomarker outcomes

We calculated greenspace exposure by averaging 2017 NDVI for a 400-m buffer around the participants’ residences, which is a common and optimal buffer size that has been used in previous studies.^25,26^ NDVI was collected from Google Earth Engine using Landsat 7 Collection 1 Tier 1 data, which has a high spatial resolution of 30 m.^27^

We assessed nine metabolic biomarkers that are related to the conditions associated with metabolic syndrome. These outcomes were measured and collected during the clinical visit, which included fasting plasma glucose (mg/dl), total cholesterol (mg/dl), low-density lipoprotein (LDL) cholesterol (mg/dl), HDL cholesterol (mg/dl), triglycerides (mg/dl), hemoglobin A1c (%), systolic blood pressure (SBP) (mmHg), diastolic blood pressure (DBP) (mmHg), and waist circumference (cm). We additionally assessed metabolic syndrome (yes; no) as an outcome of interest, where having metabolic syndrome was defined as having at least three of the following criteria: an increased waist circumference (more than 102 cm for men and 88 cm for women), elevated triglycerides (greater or equal to 150 mg/dl), low HDL cholesterol (less than 40 mg/dl in men and 50 mg/dl in women), hypertension (greater than or equal to 130 mmHg SBP or 80 mmHg DBP), and impaired fasting glucose (greater than or equal to 110 mg/dl).

### Statistical analysis

G-computation was developed by Robins (1986) and can be used to identify an average causal effect through standardization. Briefly, the mean outcome (*Y*) in the exposed (*A* = 1) and in the unexposed (*A* = 0) is estimated and standardized across the prevalence of covariates *L*, as shown in the following equation: Σ E[*Y*|*A* = a, *L* = l] × Pr[*L* = l].

A detailed visualization of the steps of g-computation derived from Hernán and Robins^28^ is provided in Figure S1; http://links.lww.com/EE/A293. There are several key assumptions for g-computation that are typical under the potential outcomes framework, including exchangeability, positivity, and consistency, as well as no measurement error and no model misspecification.

Parametric g-computation was utilized to simulate hypothetical interventions that would increase or decrease NDVI exposure around the home and observe the impacts of these exposure changes on metabolic biomarkers in participants. Minimum, maximum, and decile values of NDVI were calculated from the exposure distribution in the participants. Ten interventions on NDVI exposure were simulated (e.g., 10th percentile through the maximum, considered the “exposed” groups) and compared to the minimum NDVI exposure (e.g., the “unexposed” group). Generalized linear models were utilized for the metabolic biomarkers (i.e., the nine continuous outcome variables) and a logistic regression model was utilized for metabolic syndrome (i.e., the one binary outcome variable), which adjusted for potential confounders including sex (male; female), income ($30,000 or less; $30,000–55,000; $55,000 or more), race (White; Black; Asian; Native Hawaiian, or other Pacific Islander; American Indian/Alaska Native; unknown/other), ethnicity (Hispanic; non-Hispanic), age, and education (less than high school; high school or more). Regression estimates were then used to predict the outcome values if all participants were exposed or unexposed to each of the ten intervention deciles. Risk differences (RDs) between each decile of NDVI exposure starting from 10th percentile and the reference group (minimum NDVI) were calculated from the average predicted outcomes. Bootstrapping was used to obtain the 95% confidence interval (CI) for the RDs using 1000 iterations. A sensitivity analysis was conducted to examine the model misspecification assumption. We utilized a super learner ensemble, which combined and took the weighted average of four machine learning algorithms: generalized linear model with main terms only (SL.glm), random forest (SL.randomForest), elastic net regression (SL.glmnet), and extreme gradient boosting (SL.xgboost) using 10-fold cross-validation.^29,30^ A binomial distribution was specified for the binary metabolic syndrome outcome, and 95% CIs were also obtained using bootstrapping with 1000 iterations. It is important to note that singly robust estimators (such as g-computation) used with machine learning underperform compared to doubly robust estimators with regard to bias, variance, and CI coverage;^31^ however, the purpose of this sensitivity analysis was to compare the model fit of the models used in the main analysis to those of the ensemble models and confirm whether the RD estimates and trends between NDVI exposure and metabolic biomarker outcomes were similar.

We assessed if certain subgroups had more substantial health benefits from NDVI exposure interventions. Effect modification by demographic characteristics including sex, ethnicity, income, and age (categorized as 65 years old or greater; under 65 years old) were examined. For each effect modifier, the stratum of interest (for instance, those 65 years or older) was simulated to be exposed to each of the NDVI exposure levels (e.g., 10th percentile through the maximum), while the other categories (in this case, less than 65 years old) remained exposed to their true residential NDVI exposure value (e.g., the actual average NDVI value within a 400-m buffer around the residence). An interaction term between the given effect modifier and NDVI exposure was added to each model. Cochran Q heterogeneity tests assessed if significant differences existed between the RDs for each effect modifier, where significance was defined with a conservative threshold using a *P* value for heterogeneity at 0.05.

Analysis was conducted using Stata 16.1 (StataCorp LLC, College Station, TX) and R 4.3.1 (R Core Team, Austria, Vienna), and the Super Learner package was used for the sensitivity analysis.^29^ The analytical code can be found on the following GitHub repository: https://github.com/hdscalecollab/G-computation.

## Results

Table 1 provides a summary of the demographic characteristics of the study population (N = 555). A greater proportion of participants were high school educated or higher (90.5%), had an annual income greater than $55,000 (49.2%), female (56.0%), White (70.8%), non-Hispanic (58.0%), and under the age of 65 (68.7%). About 18.9% of participants met the criteria for metabolic syndrome. Additionally, fasting plasma total cholesterol and glucose concentrations were above the normal range, whereas average LDL cholesterol, triglycerides, hemoglobin A1c, and SBP and DBP were within the normal range. Average waist circumference was also within the normal range for both male and female participants (average female waist circumference: 86.8 cm; average male waist circumference: 96.9 cm).

**Table 1.** Summary of demographic characteristics of the study population (N = 558), distribution of NDVI exposure, and biomarker outcomes

Characteristics	n (%)/mean (SD)
Demographic characteristics	
Age	58.7 (11.0)
Sex	
Female	311 (56.0%)
Male	244 (44.0%)
Income	
$30,000 or less	154 (27.7%)
$30,000–55,000	128 (23.1%)
$55,000 or more	273 (49.2%)
Race	
White	393 (70.8%)
Black	18 (3.2%)
Asian	21 (3.8%)
American Indian/Alaska Native	24 (4.3%)
Native Hawaiian or other Pacific Islander	7 (1.3%)
Unknown/other	92 (16.6%)
Ethnicity	
Non-Hispanic	322 (58.0%)
Hispanic	233 (42.0%)
Education	
Less than high school	53 (9.5%)
High school or more	502 (90.5%)
Exposure	
Normalized difference vegetation index (NDVI)	0.17 (0.1)
Fasting plasma biomarkers, waist circumference, and METS	
Glucose (mg/dl)	103.7 (27.5)
Total cholesterol (mg/dl)	188.2 (35.1)
HDL cholesterol (mg/dl)	59.0 (17.4)
LDL cholesterol (mg/dl)	107.8 (30.4)
Triglycerides (mg/dl)	107.1 (51.6)
Systolic blood pressure (mmHg)	126.7 (17.6)
Diastolic blood pressure (mmHg)	72.7 (10.1)
Hemoglobin A1c (%)	5.6 (0.9)
Waist circumference (cm)	91.2 (20.9)
Metabolic syndrome	
Yes	105 (18.9%)
No	450 (81.1%)

Frequencies and percentages are provided for categorical variables, while means and standard deviations are provided for continuous variables.

Figures S2a–S2k; http://links.lww.com/EE/A293 provide maps depicting average NDVI exposure and health outcomes at the ZIP code level. NDVI exposure tended to be higher in northern San Diego County and lower in the southern region of the county. Higher fasting glucose concentrations seemed to be more concentrated in southern San Diego, compared to other regions, while triglyceride concentrations, waist circumference, and metabolic syndrome were higher in northern San Diego. HDL cholesterol concentrations were highest in both northern and southern San Diego, particularly inland. In contrast, greater spatial heterogeneity can be seen for LDL cholesterol, total cholesterol, SBP and DBP, and hemoglobin A1c, where patterns are more difficult to identify.

**Figure 1.**
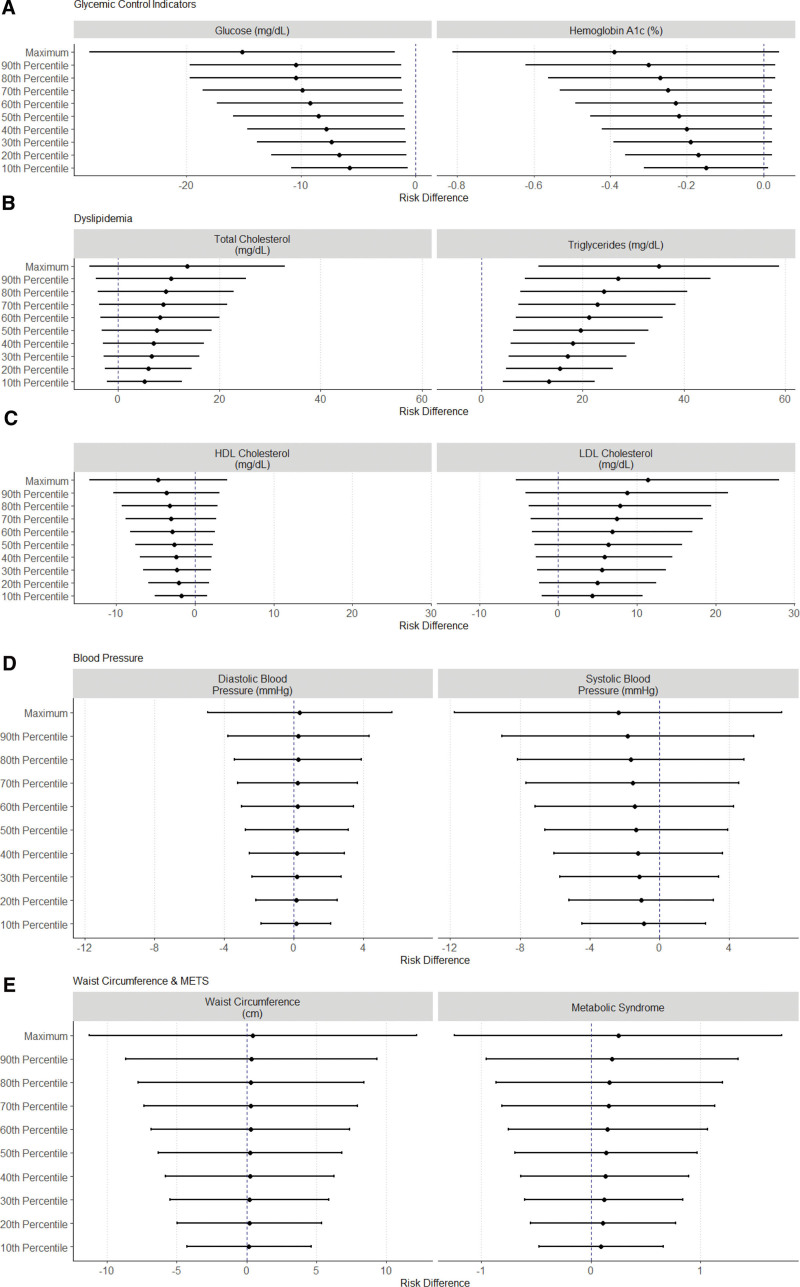
Risk differences (RDs) of average biomarker changes with simulated changes to NDVI exposure for the total population. RDs are shown as points and 95% CIs as lines on the *x*-axis and simulated NDVI exposure on the *y*-axis as deciles compared to minimum NDVI exposure for the glycemic control indicators (A: fasting glucose levels and hemoglobin A1c), dyslipidemia (B: total cholesterol and triglycerides; C: HDL cholesterol and LDL cholesterol), blood pressure (D: diastolic blood pressure and systolic blood pressure), and waist circumference and METS (E) outcomes.

**Figure 2.**
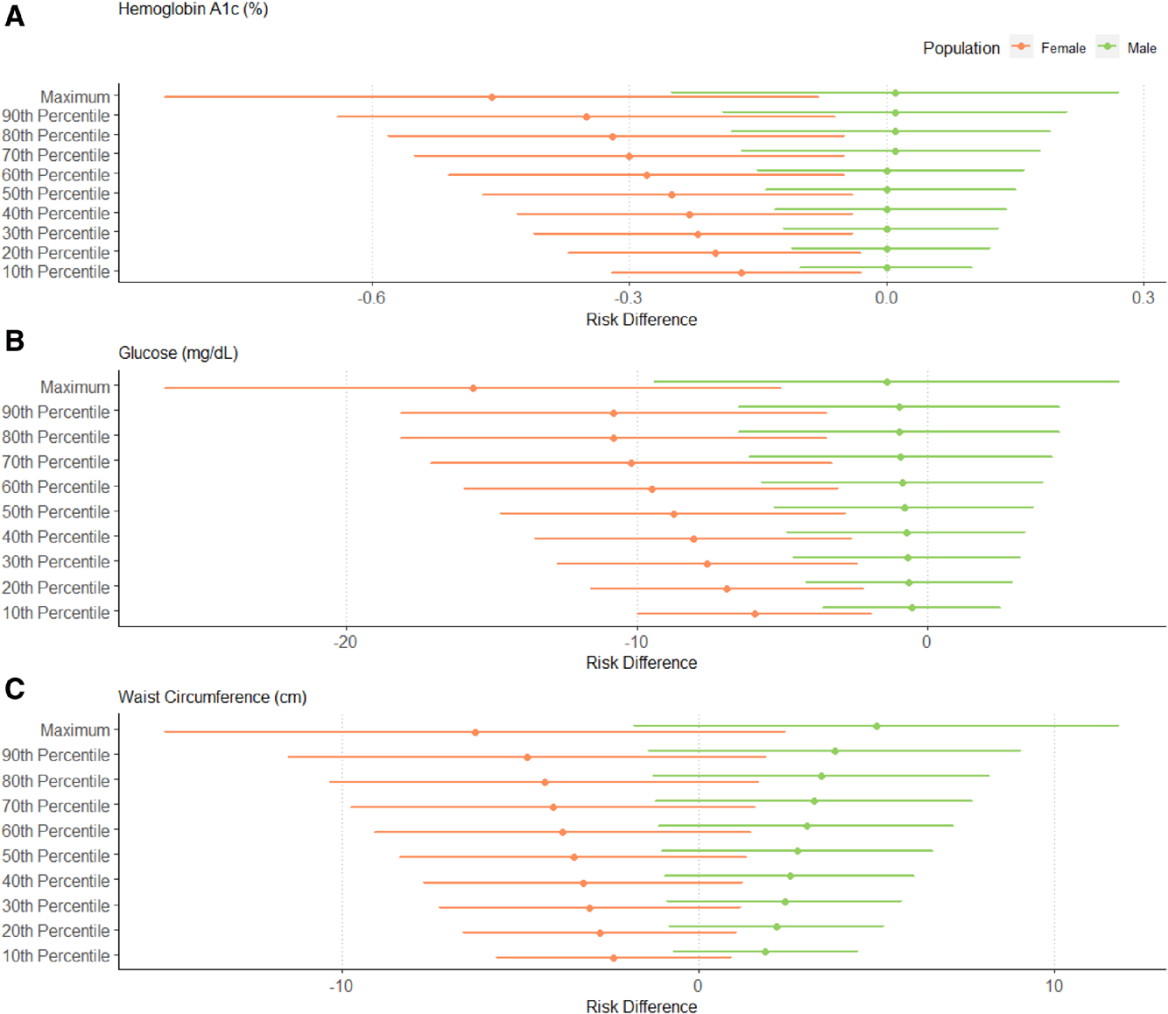
Risk differences (RDs) of average biomarker changes with simulated changes to NDVI exposure, stratified by sex. RDs are shown as points and 95% CIs as lines on the *x*-axis and simulated NDVI exposure on the *y*-axis as deciles compared to minimum NDVI exposure for hemoglobin A1c (A), glucose (B), and waist circumference (C). Effect modification by sex is depicted, where males are shown in green and females are shown in orange.

**Figure 3.**
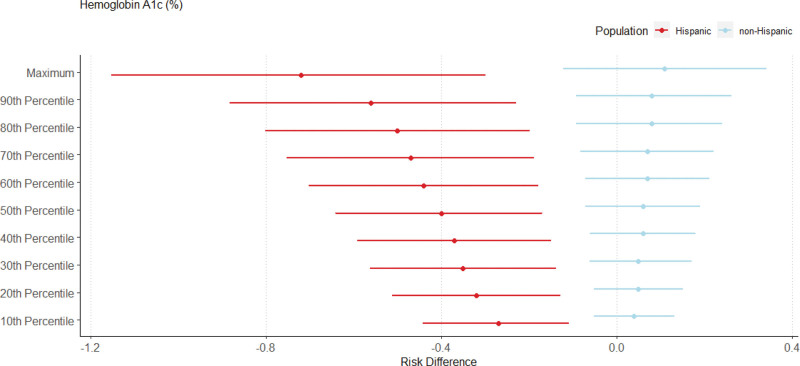
Risk differences (RDs) of average biomarker changes with simulated changes to NDVI exposure, stratified by ethnicity. RDs are shown as points and 95% CIs as lines on the *x*-axis and simulated NDVI exposure on the *y*-axis as deciles compared to minimum NDVI exposure for hemoglobin A1c. Effect modification by ethnicity is depicted, where Hispanic participants are shown in red and non-Hispanic participants are shown in blue.

**Figure 4.**
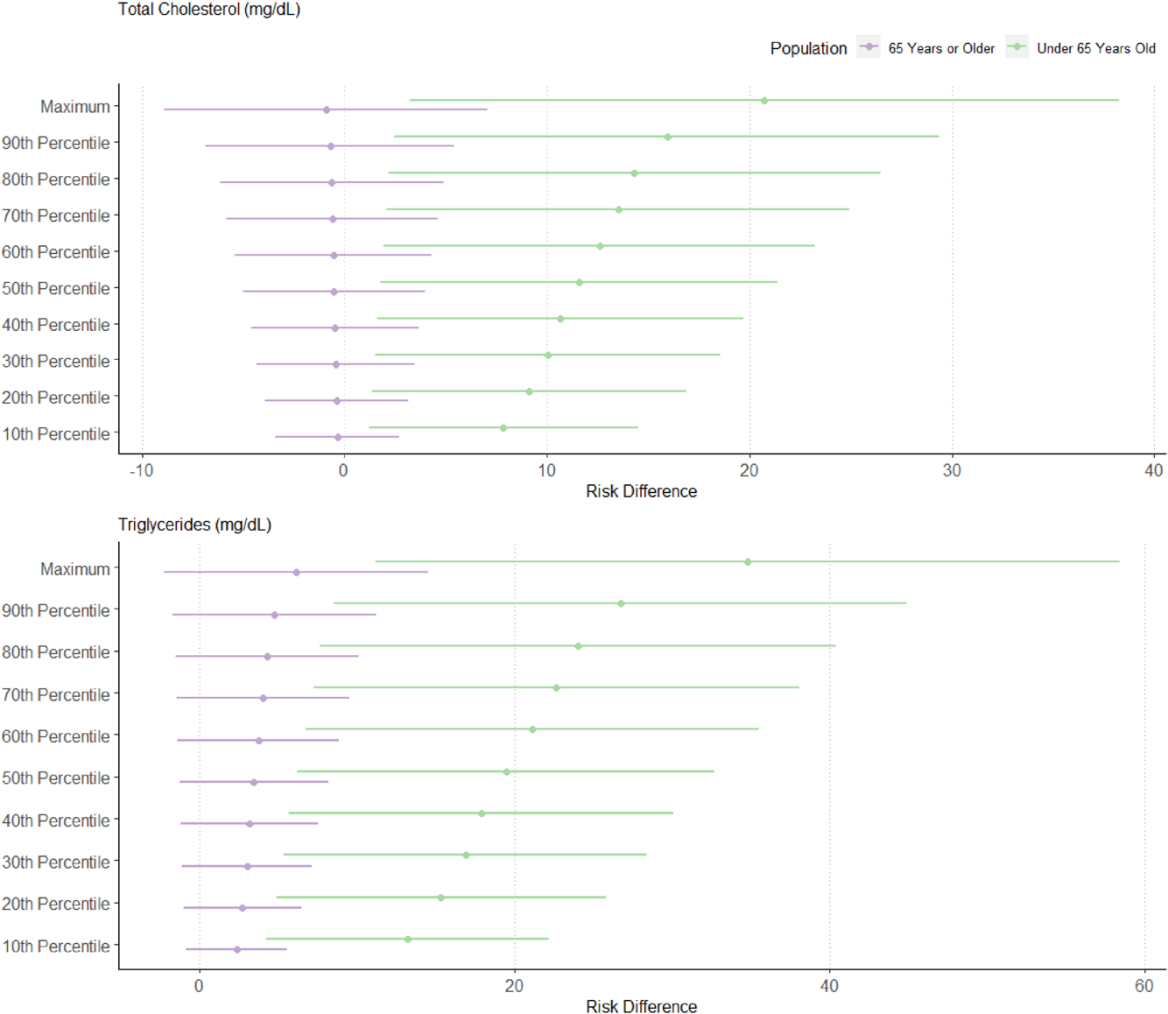
Risk differences (RDs) of average biomarker changes with simulated changes to NDVI exposure, stratified by age. RDs are shown as points and 95% CIs as lines on the *x*-axis and simulated NDVI exposure on the *y*-axis as deciles compared to minimum NDVI exposure for total cholesterol and triglycerides. Effect modification by age is depicted, where those 65 years or older are shown in purple and those under 65 years old are shown in green.

The RDs for the relationship between NDVI exposure and metabolic health outcomes from the g-computation results for the entire population are provided in Figure 1, and Figures 2–4 provide the significant effect modification results (tabulated results are provided in Tables S2–S5; http://links.lww.com/EE/A293, and all effect modification results are displayed in Figures S3–S6; http://links.lww.com/EE/A293). The following NDVI exposure groups were used: the minimum (−0.082; used as the reference group), the 10th percentile (0.087), the 20th percentile (0.114), the 30th percentile (0.134), the 40th percentile (0.147), the 50th percentile (0.166), the 60th percentile (0.188), the 70th percentile (0.208), the 80th percentile (0.225), the 90th percentile (0.260), and the maximum (0.362). For the entire population, a simulated increase in NDVI exposure led to a decrease in glucose concentrations (maximum NDVI RD: −15.16 mg/dl; 95% CI: −28.44, −1.87), hemoglobin A1c (maximum NDVI RD: −0.39%; 95% CI: −0.81, 0.04), and HDL cholesterol (maximum NDVI RD: −4.68 mg/dl; 95% CI: −13.35, 4.00). In contrast, an increase in NDVI exposure increased total cholesterol (maximum NDVI RD: 13.61 mg/dl; 95% CI: −5.59, 32.80), LDL cholesterol (maximum NDVI RD: 11.37 mg/dl; 95% CI: −5.30, 28.04), and triglyceride concentrations (maximum NDVI RD: 35.07 mg/dl; 95% CI: 11.37, 58.77). With increasing NDVI exposure, SBP (maximum NDVI RD: −2.36 mmHg; 95% CI: −11.75, 7.04), DBP (maximum NDVI RD: 0.34 mmHg; 95% CI: −4.95, 5.62), waist circumference (maximum NDVI RD: 0.46 cm; 95% CI: −11.28 12.19), and metabolic syndrome (maximum NDVI RD: 0.25; 95% CI: −1.24, 1.74) changed minimally and remained close to the null. The sensitivity analysis using the super learner ensemble is depicted in Figure S7; http://links.lww.com/EE/A293 (tabulated results are provided in Table S6; http://links.lww.com/EE/A293). Overall, a slight attenuation in the strength of the RDs can be noted for most metabolic biomarkers, including LDL cholesterol, HDL cholesterol, triglycerides, total cholesterol, glucose concentrations, hemoglobin A1c, and metabolic syndrome. The positive relationship between NDVI exposure and waist circumference and the negative relationship for SBP were stronger in the super learner model. The RDs for DBP went from slightly positive to slightly negative for the main model compared to the super learner model. With some slight but insignificant nonlinearities noted in the super learner models, most of the metabolic biomarker trends with increasing NDVI exposure tended to remain the same as the main models.

We identified significant heterogeneous effects by sex, ethnicity, and age across certain health outcomes. Differences by sex were noted for fasting plasma glucose, hemoglobin A1c, and waist circumference. For these three metabolic health outcomes, a positive, increasing relationship was identified for men with increasing NDVI exposure (glucose maximum NDVI RD: −1.40 mg/dl; 95% CI: −9.39, 6.59; hemoglobin A1c maximum NDVI RD: 0.01%; 95% CI: −0.25, 0.27; waist circumference maximum NDVI RD: 5.00; 95% CI: −1.79, 11.80), whereas a negative, decreasing relationship was identified for women (glucose maximum NDVI RD: −15.63 mg/dl; 95% CI: −26/23, −5.04; hemoglobin A1c maximum NDVI RD: −0.46%; 95% CI: −0.84, −0.08; waist circumference maximum NDVI RD: −6.26; 95% CI: −14.95, 2.44). Hispanic and non-Hispanic participants differed in their relationship between NDVI exposure and hemoglobin A1c, where a negative and decreasing relationship was observed for Hispanic participants (maximum NDVI RD: −0.72%; 95% CI: −1.15, −0.30), and a positive and slightly increasing relationship was found for non-Hispanic participants (maximum NDVI RD: 0.11%; 95% CI: −0.12, 0.34). Moreover, HDL cholesterol was on the verge of being statistically significant (*P* values ranging from 0.0500 to 0.0505), where HDL concentrations increased with increasing NDVI for Hispanic individuals (maximum NDVI RD: 3.04 mg/dl; 95% CI: −2.96, 9.04), and HDL concentrations decreased with increasing NDVI for non-Hispanic individuals (maximum NDVI RD: −5.68 mg/dl; 95% CI: −12.02, 0.65). Finally, differences by age were noted by total cholesterol and triglyceride concentrations. Increasing NDVI exposure led to a negative and slightly decreasing cholesterol as well as positive and increasing triglyceride concentrations for those 65 and older (total cholesterol maximum NDVI RD: −0.91 mg/dl; 95% CI: −8.83, 7.02; triglyceride maximum NDVI RD: 6.14 mg/dl; 95% CI: −2.18, 14.46), while a positive and increasing relationship was identified for both biomarkers for those under 65 years old (total cholesterol maximum NDVI RD: 20.74 mg/dl; 95% CI: 3.27, 38.22; triglyceride maximum NDVI RD: 34.77 mg/dl; 95% CI: 11.18, 58.36). In comparison, no significant differences by income were observed for any of the health outcomes.

## Discussion

We utilized g-computation to simulate increasing NDVI exposure levels and modeled their effects on nine metabolic biomarkers and metabolic syndrome in a diverse cohort. We found that increasing NDVI exposure led to a reduction in fasting plasma glucose, HDL cholesterol, and hemoglobin A1c and an increase in total cholesterol, LDL cholesterol, and triglyceride concentrations across all participants. In contrast, increasing NDVI exposure minimally impacted SBP, DBP, waist circumference, and metabolic syndrome. Differences in the influence of NDVI exposure on certain biomarkers were identified by sex, ethnicity, and age.

We observed that indicators of glycemic control (e.g., fasting glucose and hemoglobin A1c levels) decreased with increasing NDVI exposure. Several studies support this finding, as they identified a decrease in hemoglobin A1c^32–34^ and a decrease in fasting glucose^33,35,36^ with an increase in NDVI exposure. In contrast, contrary to our hypotheses, biomarkers related to dyslipidemia (e.g., high total cholesterol, LDL cholesterol, and triglyceride levels as well as low HDL cholesterol) increased with increasing NDVI exposure. Mixed findings have been previously identified. Yang et al^37^ and Mei et al^38^ identified a negative relationship between NDVI exposure and these blood lipids. In contrast, a study found no association between NDVI exposure and total and LDL cholesterols in children.^39^ A separate study also found close to null effects of NDVI exposure on total cholesterol and triglycerides, specifically when they assessed NDVI exposure around a 500-m buffer.^40^ Another study found a decrease in total cholesterol and an increase in LDL cholesterol with increasing NDVI exposure.^41^ Given these mixed findings, additional studies are needed to better understand the relationship between NDVI exposure and biomarkers related to dyslipidemia. Moreover, we found that SBP decreased with increasing NDVI exposure, while DBP was not strongly influenced by NDVI exposure. A systematic review identified a small protective influence of NDVI exposure on blood pressure, where SBP decreased by −0.77 mmHg and DBP decreased by −0.32 mmHg with a 0.1 unit increase in NDVI exposure.^42^ Several other studies found similar results as well.^43–45^ One study identified that greenspace exposure was protective of both high and low blood pressure.^46^ Moreover, contrary to our findings, a systematic review assessing the relationship between greenspace exposure and metabolic syndrome identified 18 papers examining this association, and they observed an odds ratio of 0.90 (95% CI: 0.87, 0.93) for the influence of 500-m NDVI on the risk of metabolic syndrome.^17^ Therefore, while our findings tend to be consistent with previous studies, such as for biomarkers related to glycemic control, more studies are needed as mixed results have been identified. Patwary et al emphasized the fact that few studies have examined the overall association between greenspace exposure and metabolic syndrome risk, making this a critical relationship to examine. Few studies have investigated the underlying mechanisms through which a greener environment influences metabolic biomarkers. One such study found that physical activity mediated the relationship between NDVI and fasting glucose,^33^ and two other studies identified that both air pollution and body mass index mediated the relationship between NDVI and biomarkers related to dyslipidemia.^37,41^ Another study identified physical activity, body mass index, and air pollution including PM_2.5_, PM_10_, nitrogen dioxide, and sulfur dioxide, as mediators in the relationship between exposure to greenness and metabolic syndrome risk.^47^ Additionally, it may be possible that increased green space improves social capital and reduces stress, which may in turn reduce the risk of metabolic syndrome.^13,48–51^ However, given the limited focus of previous literature on this topic, future work should improve understanding of the underlying mechanisms that result in greenspace impacts on these biomarkers and metabolic syndrome.

We also identified effect modification by sex, ethnicity, and age for the relationship between NDVI exposure and certain biomarkers. We found beneficial impacts of increasing NDVI exposure for women on their hemoglobin A1c, fasting glucose concentrations, and waist circumference as well as for Hispanic individuals on their hemoglobin A1c. Additionally, counter to our hypotheses, increasing NDVI exposure contributed to higher total cholesterol and triglycerides for those less than 65 years old. A few studies have assessed the effect of modification on this relationship; however, mixed results have been identified. In contrast with our study, Li et al^35^ identified a stronger negative relationship in men and older adults between NDVI exposure and fasting glucose. While we did not identify significant effect modification by sex on the relationship between NDVI and dyslipidemia-related biomarkers, inconsistent results exist, where one study identified a stronger negative relationship for men,^41^ while another identified a stronger negative relationship for women.^40^ A stronger negative relationship was also found for those with a higher education level and older adults.^37,40^ We also did not identify effect modification in the relationship between NDVI exposure and blood pressure; however, studies found higher negative relationships for men, smokers, drinkers, those exposed to lower PM_2.5_, younger individuals, and those who are overweight or obese.^43–46^ Mixed findings have been previously identified regarding NDVI exposure and dyslipidemia-related biomarkers by age. One study similarly identified an increase in NDVI led to lower HDL cholesterol and higher triglyceride levels in individuals living in London, United Kingdom, aged 45–69 years.^13^ In contrast, another study found that for every one standard deviation increase in NDVI, HDL cholesterol increased and triglycerides decreased in older adults living in Shenzhen, China.^52^ Given that few studies have assessed this relationship and mixed results have been identified, additional studies are needed to understand the impact of NDVI exposure on dyslipidemia-related biomarkers and the possible underlying mechanisms by which age plays a role in this relationship. Moreover, no studies to date have investigated the modification of this relationship between NDVI exposure and metabolic biomarkers by ethnicity. Improved understanding regarding the factors that contribute to differential associations by subgroups is necessary. It is possible that social and economic disadvantage and discrimination may contribute to certain subgroups having a greater risk of metabolic syndrome or green space deprivation,^53^ making simulated increased green space more beneficial in these subgroups compared to their counterparts; however, more research is needed to better understand these differential subgroup associations. Additionally, further studies are needed to assess this relationship in other regions and to identify whether certain subpopulations benefit more greatly from increasing greenspace exposure. This has implications for potential targeted interventions, as policies may be able to prioritize subgroups that benefit most from greening strategies.

Our study illustrates the advantages of utilizing g-computation, as it flexibly estimates the difference in the outcomes if all participants were exposed to the intervention compared to all participants being unexposed to the intervention. We assessed a diverse set of hypothetical changes in NDVI exposure on metabolic biomarkers, which can be useful for potential interventions and stakeholders. We selected these specific scenarios to have a wide range of simulated NDVI exposures to understand this etiological relationship; however, it should be noted that this approach can be used to reflect any type of intervention and can be applied to other potential policy interventions. Moreover, g-computation can be utilized to simulate the differential impacts of an intervention on health outcomes in various subpopulations. A g-computation approach that considers effect modification can aid in identifying potential inequities, where a targeted intervention can be simulated on a specific subpopulation. This has implications for tailored policies, as the most effective greening interventions can be identified, and the subpopulations that benefit most from these interventions can be prioritized. Several strengths exist for this study. This study was conducted in the ethnically diverse Community of Mine sample that includes a high proportion of Hispanic individuals with several collected metabolic risk biomarkers. Furthermore, the sampling frame was specifically designed to ensure high environmental variability across the San Diego region. However, this study also has limitations. It is possible that residual or unmeasured confounding exists, which can potentially violate the exchangeability and positivity assumptions needed to causally interpret the g-computation findings. One such example of residual confounding is structural racism and discrimination targeted toward marginalized and underrepresented populations, which are not controlled for by solely adjusting on race and ethnicity.^54–56^ Moreover, we used static NDVI exposure, where NDVI was averaged using a 400-m buffer around the residence. First, a range of buffer sizes have been used in the literature, such as those within a 500-m buffer. It may be worthwhile to explore whether differences in the associations exist based on the utilized buffer size. Second, while this static metric using such a buffer has previously been shown to be important to examine, the risk of misclassification and measurement error (another assumption needed for g-computation) is reduced when considering dynamic NDVI exposure that incorporates participants’ mobility and daily trajectories. It is important to note, however, that violations to the other g-computation assumptions, including consistency, measurement error (with regards to the outcomes), and model misspecification, are minimal, as a result of our use of well-defined NDVI exposures, the outcomes being measured and collected during a clinical visit, and our sensitivity analysis confirming similar results between our main models and our super learner ensemble models. This study was also performed in a relatively small and geographically specific sample, with limited diversity with regard to race and education, which may impact the generalizability of these findings. This sample of participants was also older, more female, more educated, more ethnically diverse, had a different racial composition (e.g., a greater proportion of American Indian/Alaska Native individuals and a lower proportion of non-Hispanic Black and non-Hispanic Asian individuals), and had a similar income composition compared to San Diego County, which may also have consequences on the generalizability of these findings.^57^ Thus, future studies should continue to evaluate the public health implications of NDVI exposure, simulate the impact of dynamic NDVI exposure on metabolic biomarkers using g-computation, and assess this relationship in other populations or regions, particularly in racially diverse groups.

## Conclusions

In this study, we simulated the impact of greenspace interventions on several metabolic biomarkers and metabolic syndrome in a population living in San Diego County. Increasing NDVI exposure was found to lower hemoglobin A1c, fasting plasma glucose, and HDL cholesterol concentrations and increase total cholesterol, LDL cholesterol, HDL cholesterol, and triglyceride concentrations across all participants. Moreover, modification of this relationship by sex, ethnicity, and age was identified. Specifically, increasing NDVI exposure was found to reduce hemoglobin A1c and fasting plasma glucose for women but not men, hemoglobin A1c for Hispanic but not non-Hispanic participants, and increasing NDVI exposure increased total cholesterol and triglycerides for participants under 65 but not participants 65 years and older. By simulating these targeted interventions, the greening strategies with the most health benefits can be identified and the subpopulations that benefit most from these interventions can be prioritized.

## Conflicts of interest statement

The authors declare that they have no conflicts of interest with regard to the content of this report.

## Acknowledgments

We thank Anna Dimitrova for her guidance on data visualization.

## Supplementary Material
