# Supplementary material for: Simulating the impact of greenspace exposure on metabolic biomarkers in a diverse population living in San Diego, California: A g-computation application
Source: Environ Epidemiol. 2024 Aug 7;8(4):e326. doi: 10.1097/EE9.0000000000000326 (PMC11309718; doi:10.1097/EE9.0000000000000326)
Supplement: Supplementary file 1 [file ee9-8-e326-s001.pdf]

Table S1. Descriptive statistics regarding the missingness for included covariates, exposure, and outcomes from the Community of Mine study participants (n=602).[1]

| Variables | Total Community of Mine Study Participants (n=602) |
|---|---|
| Sex | 0 (0.0%) |
| Income | 23 (3.8%) |
| Race | 0 (0.0%) |
| Ethnicity | 0 (0.0%) |
| Age | 0 (0.0%) |
| Education | 4 (0.7%) |
| NDVI | 0 (0.0%) |
| Fasting plasma glucose | 4 (0.7%) |
| Total cholesterol | 7 (1.2%) |
| LDL cholesterol | 16 (2.7%) |
| HDL cholesterol | 7 (1.2%) |
| Triglycerides | 7 (1.2%) |
| Hemoglobin A1c | 6 (1.0%) |
| SBP | 0 (0.0%) |
| DBP | 0 (0.0%) |
| Waist circumference | 4 (0.7%) |
| **Total number of participants included in analysis: 555 (92.2% retention)** | |

[1]Note that some participants were missing information for more than one covariate, outcome, or exposure variable.

**Table S2.** Risk differences (RDs) and 95% confidence intervals (CIs) comparing each NDVI exposure decile to minimum NDVI exposure for the dyslipedemia outcomes, including LDL cholesterol, total cholesterol, triglycerides, and HDL cholesterol for the entire study population as well as stratified by effect modifier (sex, ethnicity, income, and age). Effect modifiers are bolded if Cochran's Q heterogeneity test was found to be statistically significant (p-value < 0.05).

| | Entire Population | Male only | Female only | Hispanic only | Non-Hispanic only | Income <$30k only | Income $30-55k only | Income $55k+ only | Age 65+ yo only | Age <65 yo only |
|---|---|---|---|---|---|---|---|---|---|---|
| **LDL Cholesterol** | | | | | | | | | | |
| RD 10th - Min | 4.31 (-2.01, 10.62) | 2.34 (-1.60, 6.27) | 1.81 (-2.99, 6.62) | 1.77 (-2.84, 6.38) | 2.52 (-2.05, 7.09) | 0.65 (-2.80, 4.10) | 4.06 (0.09, 8.03) | 1.00 (-2.72, 4.72) | -0.03 (-2.78, 2.72) | 5.99 (0.33, 11.66) |
| RD 20th - Min | 5.01 (-2.33, 12.36) | 2.72 (-1.86, 7.29) | 2.11 (-3.48, 7.70) | 2.06 (-3.31, 7.42) | 2.94 (-2.38, 8.25) | 0.75 (-3.26, 4.76) | 4.72 (0.10, 9.34) | 1.16 (-3.16, 5.49) | -0.03 (-3.23, 3.17) | 6.97 (0.38, 13.56) |
| RD 30th - Min | 5.52 (-2.57, 13.60) | 2.99 (-2.05, 8.03) | 2.32 (-3.83, 8.48) | 2.26 (-3.64, 8.17) | 3.23 (-2.62, 9.08) | 0.83 (-3.59, 5.24) | 5.20 (0.11, 10.28) | 1.28 (-3.48, 6.04) | -0.03 (-3.56, 3.49) | 7.67 (0.42, 14.93) |
| RD 40th - Min | 5.85 (-2.72, 14.42) | 3.17 (-2.17, 8.51) | 2.46 (-4.07, 8.99) | 2.40 (-3.86, 8.66) | 3.43 (-2.78, 9.63) | 0.88 (-3.80, 5.56) | 5.51 (0.12, 10.90) | 1.36 (-3.69, 6.41) | -0.04 (-3.77, 3.70) | 8.14 (0.45, 15.83) |
| RD 50th - Min | 6.36 (-2.96, 15.67) | 3.45 (-2.36, 9.25) | 2.68 (-4.42, 9.77) | 2.61 (-4.19, 9.41) | 3.72 (-3.02, 10.47) | 0.95 (-4.13, 6.04) | 5.99 (0.13, 11.84) | 1.48 (-4.01, 6.96) | -0.04 (-4.10, 4.02) | 8.84 (0.48, 17.20) |
| RD 60th - Min | 6.90 (-3.21, 17.02) | 3.74 (-2.56, 10.05) | 2.91 (-4.80, 10.61) | 2.83 (-4.55, 10.22) | 4.04 (-3.28, 11.37) | 1.04 (-4.49, 6.56) | 6.50 (0.14, 12.86) | 1.60 (-4.36, 7.56) | -0.04 (-4.45, 4.37) | 9.60 (0.53, 18.68) |
| RD 70th - Min | 7.41 (-3.45, 18.27) | 4.02 (-2.75, 10.78) | 3.12 (-5.15, 11.39) | 3.04 (-4.89, 10.97) | 4.34 (-3.52, 12.20) | 1.11 (-4.82, 7.04) | 6.98 (0.15, 13.81) | 1.72 (-4.68, 8.12) | -0.05 (-4.78, 4.69) | 10.31 (0.56, 20.05) |
| RD 80th - Min | 7.86 (-3.66, 19.38) | 4.26 (-2.92, 11.44) | 3.31 (-5.46, 12.08) | 3.22 (-5.18, 11.63) | 4.60 (-3.73, 12.94) | 1.18 (-5.11, 7.47) | 7.40 (0.16, 14.64) | 1.82 (-4.96, 8.61) | -0.05 (-5.07, 4.97) | 10.93 (0.60, 21.26) |
| RD 90th - Min | 8.74 (-4.07, 21.54) | 4.74 (-3.24, 12.72) | 3.68 (-6.07, 13.43) | 3.59 (-5.76, 12.93) | 5.12 (-4.15, 14.39) | 1.31 (-5.68, 8.31) | 8.23 (0.18, 16.28) | 2.03 (-5.52, 9.57) | -0.05 (-5.63, 5.53) | 12.15 (0.67, 23.64) |
| RD Max - Min | 11.37 (-5.30, 28.04) | 6.17 (-4.22, 16.55) | 4.79 (-7.91, 17.48) | 4.67 (-7.50, 16.84) | 6.66 (-5.40, 18.73) | 1.71 (-7.40, 10.81) | 10.71 (0.23, 21.19) | 2.64 (-7.18, 12.46) | -0.07 (-7.33, 7.19) | 15.82 (0.87, 30.77) |
| **Total Cholesterol** | | | | | | | | | | |
| RD 10th - Min | 5.15 (-2.12, 12.43) | 2.83 (-1.66, 7.32) | 2.13 (-3.37, 7.62) | 4.05 (-1.38, 9.47) | 1.87 (-3.28, 7.03) | 1.60 (-2.60, 5.81) | 2.92 (-1.36, 7.20) | 1.59 (-2.72, 5.90) | **-0.34 (-3.35, 2.66)** | **7.86 (1.24, 14.48)** |
| RD 20th - Min | 6.00 (-2.46, 14.45) | 3.29 (-1.93, 8.51) | 2.47 (-3.91, 8.86) | 4.71 (-1.61, 11.02) | 2.18 (-3.81, 8.17) | 1.86 (-3.03, 6.75) | 3.40 (-1.58, 8.38) | 1.85 (-3.17, 6.86) | **-0.40 (-3.89, 3.09)** | **9.14 (1.44, 16.84)** |
| RD 30th - Min | 6.60 (-2.71, 15.91) | 3.62 (-2.13, 9.37) | 2.72 (-4.31, 9.75) | 5.18 (-1.77, 12.13) | 2.40 (-4.20, 9.00) | 2.05 (-3.33, 7.43) | 3.74 (-1.74, 9.23) | 2.04 (-3.49, 7.56) | **-0.44 (-4.28, 3.40)** | **10.06 (1.59, 18.54)** |
| RD 40th - Min | 7.00 (-2.87, 16.87) | 3.84 (-2.25, 9.94) | 2.89 (-4.57, 10.34) | 5.49 (-1.88, 12.86) | 2.54 (-4.45, 9.54) | 2.18 (-3.53, 7.88) | 3.97 (-1.84, 9.78) | 2.16 (-3.70, 8.01) | **-0.47 (-4.54, 3.61)** | **10.67 (1.68, 19.65)** |
| RD 50th - Min | 7.60 (-3.12, 18.33) | 4.17 (-2.45, 10.80) | 3.13 (-4.96, 11.23) | 5.97 (-2.04, 13.97) | 2.76 (-4.84, 10.37) | 2.36 (-3.84, 8.57) | 4.31 (-2.00, 10.63) | 2.34 (-4.02, 8.70) | **-0.51 (-4.94, 3.92)** | **11.59 (1.83, 21.36)** |
| RD 60th - Min | 8.26 (-3.39, 19.91) | 4.53 (-2.66, 11.73) | 3.41 (-5.39, 12.20) | 6.48 (-2.22, 15.18) | 3.00 (-5.25, 11.26) | 2.57 (-4.17, 9.31) | 4.69 (-2.18, 11.55) | 2.55 (-4.36, 9.46) | **-0.55 (-5.36, 4.26)** | **12.59 (1.98, 23.20)** |
| RD 70th - Min | 8.87 (-3.64, 21.37) | 4.86 (-2.86, 12.59) | 3.65 (-5.79, 13.10) | 6.96 (-2.38, 16.29) | 3.22 (-5.64, 12.08) | 2.76 (-4.47, 9.99) | 5.03 (-2.33, 12.39) | 2.73 (-4.68, 10.15) | **-0.59 (-5.75, 4.57)** | **13.51 (2.13, 24.90)** |
| RD 80th - Min | 9.40 (-3.86, 22.66) | 5.16 (-3.03, 13.35) | 3.88 (-6.14, 13.89) | 7.38 (-2.52, 17.28) | 3.42 (-5.98, 12.82) | 2.92 (-4.74, 10.59) | 5.33 (-2.48, 13.14) | 2.90 (-4.96, 10.76) | **-0.63 (-6.10, 4.85)** | **14.33 (2.26, 26.40)** |
| RD 90th - Min | 10.45 (-4.29, 25.20) | 5.74 (-3.37, 14.84) | 4.31 (-6.82, 15.44) | 8.20 (-2.80, 19.21) | 3.80 (-6.65, 14.25) | 3.25 (-5.27, 11.78) | 5.93 (-2.75, 14.61) | 3.22 (-5.52, 11.97) | **-0.70 (-6.79, 5.39)** | **15.93 (2.51, 29.36)** |
| RD Max - Min | 13.61 (-5.59, 32.80) | 7.47 (-4.38, 19.32) | 5.61 (-8.88, 20.10) | 10.68 (-3.65, 25.01) | 4.95 (-8.65, 18.55) | 4.23 (-6.86, 15.33) | 7.72 (-3.58, 19.02) | 4.20 (-7.19, 15.58) | **-0.91 (-8.83, 7.02)** | **20.74 (3.27, 38.22)** |
| **Triglycerides** | | | | | | | | | | |
| RD 10th - Min | 13.29 (4.31, 22.26) | 7.07 (1.95, 12.20) | 5.77 (-2.03, 13.57) | 5.78 (-2.17, 13.72) | 7.59 (1.90, 13.28) | 5.45 (-1.72, 12.62) | 7.05 (1.17, 12.93) | 3.48 (-1.38, 8.34) | **2.33 (-0.82, 5.48)** | **13.17 (4.24, 22.11)** |
| RD 20th - Min | 15.45 (5.01, 25.90) | 8.23 (2.27, 14.19) | 6.71 (-2.36, 15.79) | 6.72 (-2.52, 15.96) | 8.83 (2.20, 15.45) | 6.34 (-2.00, 14.68) | 8.20 (1.36, 15.04) | 4.05 (-1.61, 9.70) | **2.71 (-0.96, 6.37)** | **15.32 (4.93, 25.72)** |
| RD 30th - Min | 17.01 (5.52, 28.50) | 9.06 (2.50, 15.62) | 7.39 (-2.60, 17.38) | 7.40 (-2.77, 17.57) | 9.72 (2.43, 17.01) | 6.98 (-2.20, 16.16) | 9.03 (1.49, 16.56) | 4.45 (-1.77, 10.68) | **2.98 (-1.06, 7.01)** | **16.87 (5.42, 28.31)** |
| RD 40th - Min | 18.04 (5.85, 30.22) | 9.60 (2.65, 16.56) | 7.83 (-2.76, 18.42) | 7.84 (-2.94, 18.63) | 10.30 (2.57, 18.03) | 7.40 (-2.34, 17.13) | 9.57 (1.58, 17.56) | 4.72 (-1.88, 11.32) | **3.16 (-1.12, 7.44)** | **17.88 (5.75, 30.01)** |
| RD 50th - Min | 19.60 (6.36, 32.84) | 10.44 (2.88, 18.00) | 8.51 (-3.00, 20.02) | 8.52 (-3.20, 20.24) | 11.19 (2.80, 19.59) | 8.04 (-2.54, 18.61) | 10.40 (1.72, 19.08) | 5.13 (-2.04, 12.30) | **3.43 (-1.22, 8.08)** | **19.43 (6.25, 32.61)** |
| RD 60th - Min | 21.29 (6.90, 35.68) | 11.34 (3.12, 19.55) | 9.25 (-3.26, 21.75) | 9.26 (-3.47, 21.99) | 12.16 (3.04, 21.28) | 8.73 (-2.76, 20.22) | 11.30 (1.87, 20.72) | 5.57 (-2.21, 13.36) | **3.73 (-1.32, 8.78)** | **21.11 (6.79, 35.43)** |
| RD 70th - Min | 22.85 (7.41, 38.29) | 12.17 (3.35, 20.98) | 9.92 (-3.49, 23.34) | 9.94 (-3.73, 23.60) | 13.05 (3.26, 22.84) | 9.37 (-2.96, 21.70) | 12.12 (2.01, 22.24) | 5.98 (-2.38, 14.34) | **4.00 (-1.42, 9.42)** | **22.65 (7.29, 38.02)** |
| RD 80th - Min | 24.23 (7.86, 40.61) | 12.90 (3.56, 22.25) | 10.52 (-3.70, 24.75) | 10.54 (-3.95, 25.03) | 13.84 (3.46, 24.23) | 9.94 (-3.14, 23.02) | 12.86 (2.13, 23.59) | 6.34 (-2.52, 15.21) | **4.24 (-1.50, 9.99)** | **24.03 (7.73, 40.32)** |
| RD 90th - Min | 26.94 (8.74, 45.15) | 14.35 (3.95, 24.74) | 11.70 (-4.12, 27.52) | 11.72 (-4.39, 27.83) | 15.39 (3.84, 26.93) | 11.05 (-3.49, 25.59) | 14.30 (2.37, 26.23) | 7.05 (-2.80, 16.91) | **4.72 (-1.67, 11.11)** | **26.71 (8.59, 44.83)** |
| RD Max - Min | 35.07 (11.37, 58.77) | 18.68 (5.15, 32.21) | 15.23 (-5.36, 35.82) | 15.25 (-5.72, 36.23) | 20.03 (5.00, 35.06) | 14.38 (-4.54, 33.31) | 18.61 (3.08, 34.14) | 9.18 (-3.65, 22.01) | **6.14 (-2.18, 14.46)** | **34.77 (11.18, 58.36)** |
| **HDL Cholesterol** | | | | | | | | | | |
| RD 10th - Min | -1.77 (-5.06, 1.51) | -0.93 (-2.80, 0.95) | -0.79 (-3.80, 2.22) | 1.15 (-1.12, 3.42) | -2.15 (-4.55, 0.25) | -0.15 (-1.68, 1.39) | -2.53 (-4.62, -0.43) | -0.08 (-2.28, 2.13) | -0.75 (-2.01, 0.51) | -0.79 (-3.91, 2.33) |
| RD 20th - Min | -2.06 (-5.88, 1.76) | -1.08 (-3.26, 1.10) | -0.92 (-4.42, 2.58) | 1.34 (-1.31, 3.98) | -2.50 (-5.30, 0.29) | -0.17 (-1.96, 1.62) | -2.94 (-5.37, -0.51) | -0.09 (-2.66, 2.48) | -0.87 (-2.33, 0.59) | -0.92 (-4.55, 2.71) |
| RD 30th - Min | -2.27 (-6.48, 1.94) | -1.19 (-3.59, 1.21) | -1.01 (-4.87, 2.84) | 1.47 (-1.44, 4.38) | -2.76 (-5.83, 0.32) | -0.19 (-2.16, 1.78) | -3.24 (-5.91, -0.56) | -0.10 (-2.92, 2.73) | -0.96 (-2.57, 0.65) | -1.01 (-5.01, 2.98) |
| RD 40th - Min | -2.41 (-6.87, 2.06) | -1.26 (-3.80, 1.29) | -1.08 (-5.16, 3.01) | 1.56 (-1.52, 4.65) | -2.92 (-6.18, 0.34) | -0.20 (-2.29, 1.89) | -3.43 (-6.27, -0.59) | -0.10 (-3.10, 2.89) | -1.02 (-2.72, 0.69) | -1.07 (-5.31, 3.16) |
| RD 50th - Min | -2.61 (-7.46, 2.23) | -1.37 (-4.13, 1.40) | -1.17 (-5.61, 3.27) | 1.70 (-1.66, 5.05) | -3.17 (-6.72, 0.37) | -0.22 (-2.48, 2.05) | -3.73 (-6.81, -0.64) | -0.11 (-3.37, 3.15) | -1.10 (-2.96, 0.75) | -1.17 (-5.77, 3.44) |
| RD 60th - Min | -2.84 (-8.11, 2.43) | -1.48 (-4.49, 1.52) | -1.27 (-6.09, 3.55) | 1.84 (-1.80, 5.49) | -3.45 (-7.29, 0.40) | -0.23 (-2.70, 2.23) | -4.05 (-7.40, -0.70) | -0.12 (-3.66, 3.42) | -1.20 (-3.21, 0.82) | -1.27 (-6.27, 3.73) |
| RD 70th - Min | -3.05 (-8.70, 2.60) | -1.59 (-4.82, 1.63) | -1.36 (-6.54, 3.81) | 1.98 (-1.93, 5.89) | -3.70 (-7.83, 0.43) | -0.25 (-2.90, 2.39) | -4.35 (-7.94, -0.75) | -0.13 (-3.93, 3.67) | -1.29 (-3.45, 0.88) | -1.36 (-6.73, 4.01) |
| RD 80th - Min | -3.23 (-9.23, 2.76) | -1.69 (-5.11, 1.73) | -1.45 (-6.93, 4.04) | 2.10 (-2.05, 6.25) | -3.93 (-8.30, 0.45) | -0.27 (-3.07, 2.54) | -4.61 (-8.43, -0.79) | -0.14 (-4.17, 3.89) | -1.36 (-3.66, 0.93) | -1.44 (-7.13, 4.25) |
| RD 90th - Min | -3.59 (-10.26, 3.07) | -1.88 (-5.68, 1.92) | -1.61 (-7.71, 4.49) | 2.33 (-2.28, 6.94) | -4.36 (-9.23, 0.50) | -0.30 (-3.41, 2.82) | -5.12 (-9.37, -0.88) | -0.15 (-4.63, 4.32) | -1.52 (-4.07, 1.03) | -1.60 (-7.93, 4.72) |
| RD Max - Min | -4.68 (-13.35, 4.00) | -2.45 (-7.39, 2.50) | -2.09 (-10.03, 5.85) | 3.04 (-2.96, 9.04) | -5.68 (-12.02, 0.65) | -0.39 (-4.45, 3.67) | -6.67 (-12.20, -1.15) | -0.20 (-6.03, 5.63) | -1.98 (-5.30, 1.34) | -2.09 (-10.33, 6.15) |

**Table S3.** Risk differences (RDs) and 95% confidence intervals (CIs) comparing each NDVI exposure decile to minimum NDVI exposure for the glycemic control indicators, including blood glucose levels and hemoglobin A1C for the entire study population as well as stratified by effect modifier (sex, ethnicity, income, and age). Effect modifiers are bolded if Cochran's Q heterogeneity test was found to be statistically significant (p-value < 0.05).

| | Entire Population | Male only | Female only | Hispanic only | Non-Hispanic only | Income <$30k only | Income $30-55k only | Income $55k+ only | **Age 65+ yo only** | **Age <65 yo only** |
|---|---|---|---|---|---|---|---|---|---|---|
| **Glucose** | | | | | | | | | | |
| RD 10th - Min | -5.74 (-10.77, -0.71) | **-0.53 (-3.56, 2.50)** | **-5.92 (-9.94, -1.91)** | -5.86 (-10.66, -1.07) | -1.28 (-4.23, 1.66) | -2.00 (-5.22, 1.23) | -0.64 (-3.59, 2.30) | -2.89 (-5.91, 0.13) | -1.32 (-3.15, 0.52) | -5.01 (-9.21, -0.81) |
| RD 20th - Min | -6.68 (-12.53, -0.83) | **-0.62 (-4.14, 2.90)** | **-6.89 (-11.56, -2.22)** | -6.82 (-12.40, -1.24) | -1.49 (-4.92, 1.94) | -2.32 (-6.07, 1.43) | -0.75 (-4.17, 2.68) | -3.36 (-6.87, 0.15) | -1.53 (-3.67, 0.60) | -5.83 (-10.71, -0.94) |
| RD 30th - Min | -7.35 (-13.80, -0.91) | **-0.68 (-4.56, 3.20)** | **-7.58 (-12.72, -2.44)** | -7.51 (-13.65, -1.37) | -1.64 (-5.41, 2.13) | -2.56 (-6.69, 1.58) | -0.82 (-4.59, 2.95) | -3.70 (-7.57, 0.16) | -1.69 (-4.04, 0.66) | -6.41 (-11.79, -1.04) |
| RD 40th - Min | -7.80 (-14.63, -0.96) | **-0.72 (-4.83, 3.39)** | **-8.04 (-13.49, -2.59)** | -7.96 (-14.48, -1.45) | -1.74 (-5.74, 2.26) | -2.71 (-7.09, 1.67) | -0.87 (-4.87, 3.12) | -3.93 (-8.02, 0.17) | -1.79 (-4.28, 0.70) | -6.80 (-12.50, -1.10) |
| RD 50th - Min | -8.47 (-15.89, -1.05) | **-0.78 (-5.25, 3.68)** | **-8.74 (-14.66, -2.82)** | -8.65 (-15.73, -1.57) | -1.89 (-6.24, 2.45) | -2.94 (-7.70, 1.81) | -0.95 (-5.29, 3.40) | -4.27 (-8.72, 0.18) | -1.94 (-4.65, 0.77) | -7.39 (-13.58, -1.19) |
| RD 60th - Min | -9.20 (-17.27, -1.14) | **-0.85 (-5.70, 4.00)** | **-9.49 (-15.92, -3.06)** | -9.40 (-17.09, -1.71) | -2.05 (-6.77, 2.67) | -3.20 (-8.37, 1.97) | -1.03 (-5.75, 3.69) | -4.63 (-9.47, 0.20) | -2.11 (-5.05, 0.83) | -8.03 (-14.75, -1.30) |
| RD 70th - Min | -9.88 (-18.53, -1.22) | **-0.91 (-6.12, 4.29)** | **-10.19 (-17.09, -3.28)** | -10.09 (-18.34, -1.83) | -2.20 (-7.27, 2.86) | -3.43 (-8.98, 2.12) | -1.10 (-6.17, 3.96) | -4.97 (-10.16, 0.22) | -2.26 (-5.42, 0.89) | -8.61 (-15.83, -1.39) |
| RD 80th - Min | -10.47 (-19.65, -1.29) | **-0.97 (-6.49, 4.55)** | **-10.80 (-18.12, -3.48)** | -10.70 (-19.45, -1.95) | -2.34 (-7.71, 3.04) | -3.64 (-9.53, 2.24) | -1.17 (-6.54, 4.20) | -5.28 (-10.78, 0.23) | -2.40 (-5.75, 0.95) | -9.14 (-16.79, -1.48) |
| RD 90th - Min | -10.47 (-19.65, -1.29) | **-0.97 (-6.49, 4.55)** | **-10.80 (-18.12, -3.48)** | -10.70 (-19.45, -1.95) | -2.34 (-7.71, 3.04) | -3.64 (-9.53, 2.24) | -1.17 (-6.54, 4.20) | -5.28 (-10.78, 0.23) | -2.40 (-5.75, 0.95) | -9.14 (-16.79, -1.48) |
| RD Max - Min | -15.16 (-28.44, -1.87) | **-1.40 (-9.39, 6.59)** | **-15.63 (-26.23, -5.04)** | -15.48 (-28.15, -2.82) | -3.38 (-11.16, 4.39) | -5.27 (-13.79, 3.25) | -1.70 (-9.47, 6.08) | -7.63 (-15.60, 0.33) | -3.47 (-8.32, 1.37) | -13.22 (-24.31, -2.14) |
| **Hemoglobin A1C** | | | | | | | | | | |
| RD 10th - Min | -0.15 (-0.31, 0.01) | 0.00 (-0.10, 0.10) | -0.17 (-0.32, -0.03) | **-0.27 (-0.44, -0.11)** | **0.04 (-0.05, 0.13)** | -0.14 (-0.26, -0.02) | 0.03 (-0.09, 0.15) | -0.04 (-0.12, 0.04) | -0.02 (-0.08, 0.04) | -0.16 (-0.31, 0.00) |
| RD 20th - Min | -0.17 (-0.36, 0.02) | 0.00 (-0.11, 0.12) | -0.20 (-0.37, -0.03) | **-0.32 (-0.51, -0.13)** | **0.05 (-0.05, 0.15)** | -0.17 (-0.31, -0.03) | 0.04 (-0.10, 0.18) | -0.04 (-0.14, 0.05) | -0.02 (-0.09, 0.05) | -0.18 (-0.36, -0.01) |
| RD 30th - Min | -0.19 (-0.39, 0.02) | 0.00 (-0.12, 0.13) | -0.22 (-0.41, -0.04) | **-0.35 (-0.56, -0.14)** | **0.05 (-0.06, 0.17)** | -0.18 (-0.34, -0.03) | 0.04 (-0.11, 0.20) | -0.05 (-0.15, 0.05) | -0.02 (-0.10, 0.05) | -0.20 (-0.40, -0.01) |
| RD 40th - Min | -0.20 (-0.42, 0.02) | 0.00 (-0.13, 0.14) | -0.23 (-0.43, -0.04) | **-0.37 (-0.59, -0.15)** | **0.06 (-0.06, 0.18)** | -0.20 (-0.36, -0.03) | 0.05 (-0.12, 0.21) | -0.05 (-0.16, 0.06) | -0.03 (-0.11, 0.05) | -0.22 (-0.43, -0.01) |
| RD 50th - Min | -0.22 (-0.45, 0.02) | 0.00 (-0.14, 0.15) | -0.25 (-0.47, -0.04) | **-0.40 (-0.64, -0.17)** | **0.06 (-0.07, 0.19)** | -0.21 (-0.39, -0.04) | 0.05 (-0.13, 0.23) | -0.06 (-0.17, 0.06) | -0.03 (-0.11, 0.06) | -0.23 (-0.46, -0.01) |
| RD 60th - Min | -0.23 (-0.49, 0.02) | 0.00 (-0.15, 0.16) | -0.28 (-0.51, -0.05) | **-0.44 (-0.70, -0.18)** | **0.07 (-0.07, 0.21)** | -0.23 (-0.42, -0.04) | 0.06 (-0.14, 0.25) | -0.06 (-0.19, 0.07) | -0.03 (-0.12, 0.06) | -0.25 (-0.50, -0.01) |
| RD 70th - Min | -0.25 (-0.53, 0.02) | **0.01 (-0.17, 0.18)** | **-0.30 (-0.55, -0.05)** | **-0.47 (-0.75, -0.19)** | **0.07 (-0.08, 0.22)** | -0.25 (-0.46, -0.04) | 0.06 (-0.15, 0.27) | -0.06 (-0.20, 0.07) | -0.03 (-0.13, 0.07) | -0.27 (-0.54, -0.01) |
| RD 80th - Min | -0.27 (-0.56, 0.03) | **0.01 (-0.18, 0.19)** | **-0.32 (-0.58, -0.05)** | **-0.50 (-0.80, -0.20)** | **0.08 (-0.09, 0.24)** | -0.26 (-0.48, -0.04) | 0.06 (-0.16, 0.28) | -0.07 (-0.21, 0.08) | -0.03 (-0.14, 0.07) | -0.29 (-0.57, -0.01) |
| RD 90th - Min | -0.30 (-0.62, 0.03) | **0.01 (-0.19, 0.21)** | **-0.35 (-0.64, -0.06)** | **-0.56 (-0.88, -0.23)** | **0.08 (-0.09, 0.26)** | -0.29 (-0.54, -0.05) | 0.07 (-0.17, 0.31) | -0.08 (-0.24, 0.09) | -0.04 (-0.16, 0.08) | -0.32 (-0.64, -0.01) |
| RD Max - Min | -0.39 (-0.81, 0.04) | **0.01 (-0.25, 0.27)** | **-0.46 (-0.84, -0.08)** | **-0.72 (-1.15, -0.30)** | **0.11 (-0.12, 0.34)** | -0.38 (-0.70, -0.06) | 0.09 (-0.23, 0.41) | -0.10 (-0.31, 0.11) | -0.05 (-0.21, 0.10) | -0.42 (-0.83, -0.01) |

**Table S4.** Risk differences (RDs) and 95% confidence intervals (CIs) comparing each NDVI exposure decile to minimum NDVI exposure for the hypertension outcomes, including diastolic blood pressure and systolic blood pressure for the entire study population as well as stratified by effect modifier (sex, ethnicity, income, and age). Effect modifiers are bolded if Cochran's Q heterogeneity test was found to be statistically significant (p-value < 0.05).

| | Entire Population | Male only | Female only | Hispanic only | Non-Hispanic only | Income <$30k only | Income $30-55k only | Income $55k+ only | **Age 65+ yo only** | **Age <65 yo only** |
|---|---|---|---|---|---|---|---|---|---|---|
| **Diastolic Blood Pressure** | | | | | | | | | | |
| RD 10th - Min | 0.13 (-1.87, 2.13) | 0.13 (-1.84, 2.10) | 0.13 (-1.91, 2.17) | 0.13 (-1.81, 2.06) | 0.13 (-1.85, 2.10) | 0.13 (-1.85, 2.11) | 0.13 (-1.83, 2.09) | 0.13 (-1.87, 2.13) | 0.13 (-1.83, 2.08) | 0.13 (-1.85, 2.10) |
| RD 20th - Min | 0.15 (-2.18, 2.48) | 0.15 (-2.14, 2.44) | 0.15 (-2.22, 2.52) | 0.15 (-2.10, 2.40) | 0.15 (-2.15, 2.44) | 0.15 (-2.16, 2.45) | 0.15 (-2.13, 2.43) | 0.15 (-2.18, 2.48) | 0.15 (-2.13, 2.42) | 0.15 (-2.15, 2.45) |
| RD 30th - Min | 0.16 (-2.40, 2.73) | 0.16 (-2.36, 2.68) | 0.16 (-2.45, 2.77) | 0.16 (-2.31, 2.64) | 0.16 (-2.36, 2.69) | 0.16 (-2.37, 2.70) | 0.16 (-2.35, 2.67) | 0.16 (-2.40, 2.73) | 0.16 (-2.34, 2.67) | 0.16 (-2.37, 2.69) |
| RD 40th - Min | 0.17 (-2.54, 2.89) | 0.17 (-2.50, 2.85) | 0.17 (-2.59, 2.94) | 0.17 (-2.45, 2.80) | 0.17 (-2.51, 2.85) | 0.17 (-2.52, 2.86) | 0.17 (-2.49, 2.83) | 0.17 (-2.54, 2.89) | 0.17 (-2.48, 2.83) | 0.17 (-2.51, 2.86) |
| RD 50th - Min | 0.19 (-2.76, 3.14) | 0.19 (-2.72, 3.09) | 0.19 (-2.82, 3.20) | 0.19 (-2.66, 3.04) | 0.19 (-2.72, 3.10) | 0.19 (-2.73, 3.11) | 0.19 (-2.70, 3.08) | 0.19 (-2.76, 3.14) | 0.19 (-2.70, 3.07) | 0.19 (-2.73, 3.10) |
| RD 60th - Min | 0.20 (-3.00, 3.41) | 0.20 (-2.95, 3.36) | 0.20 (-3.06, 3.47) | 0.20 (-2.89, 3.30) | 0.20 (-2.96, 3.37) | 0.20 (-2.97, 3.38) | 0.20 (-2.94, 3.35) | 0.20 (-3.00, 3.41) | 0.20 (-2.93, 3.34) | 0.20 (-2.96, 3.37) |
| RD 70th - Min | 0.22 (-3.22, 3.66) | 0.22 (-3.17, 3.60) | 0.22 (-3.29, 3.72) | 0.22 (-3.11, 3.54) | 0.22 (-3.17, 3.61) | 0.22 (-3.19, 3.63) | 0.22 (-3.15, 3.59) | 0.22 (-3.22, 3.66) | 0.22 (-3.14, 3.58) | 0.22 (-3.18, 3.62) |
| RD 80th - Min | 0.23 (-3.42, 3.88) | 0.23 (-3.36, 3.82) | 0.23 (-3.49, 3.95) | 0.23 (-3.29, 3.76) | 0.23 (-3.37, 3.83) | 0.23 (-3.38, 3.85) | 0.23 (-3.34, 3.81) | 0.23 (-3.42, 3.88) | 0.23 (-3.34, 3.80) | 0.23 (-3.37, 3.84) |
| RD 90th - Min | 0.26 (-3.80, 4.32) | 0.26 (-3.73, 4.25) | 0.26 (-3.88, 4.39) | 0.26 (-3.66, 4.18) | 0.26 (-3.74, 4.26) | 0.26 (-3.76, 4.27) | 0.26 (-3.72, 4.23) | 0.26 (-3.80, 4.32) | 0.26 (-3.71, 4.22) | 0.26 (-3.75, 4.27) |
| RD Max - Min | 0.34 (-4.95, 5.62) | 0.34 (-4.86, 5.53) | 0.34 (-5.04, 5.72) | 0.34 (-4.77, 5.44) | 0.34 (-4.87, 5.54) | 0.34 (-4.89, 5.57) | 0.34 (-4.84, 5.51) | 0.34 (-4.95, 5.62) | 0.34 (-4.83, 5.50) | 0.34 (-4.88, 5.55) |
| **Systolic Blood Pressure** | | | | | | | | | | |
| RD 10th - Min | -0.89 (-4.45, 2.67) | 0.04 (-2.18, 2.26) | -1.09 (-3.70, 1.52) | -0.41 (-2.86, 2.03) | -0.50 (-3.00, 2.01) | 0.68 (-1.19, 2.55) | -1.41 (-3.51, 0.69) | -0.46 (-2.69, 1.78) | -0.50 (-2.03, 1.04) | -0.14 (-2.89, 2.62) |
| RD 20th - Min | -1.04 (-5.18, 3.10) | 0.05 (-2.53, 2.63) | -1.27 (-4.30, 1.77) | -0.48 (-3.32, 2.36) | -0.58 (-3.49, 2.34) | 0.79 (-1.39, 2.97) | -1.64 (-4.09, 0.80) | -0.53 (-3.13, 2.07) | -0.58 (-2.36, 1.21) | -0.16 (-3.37, 3.05) |
| RD 30th - Min | -1.14 (-5.70, 3.41) | 0.05 (-2.79, 2.89) | -1.40 (-4.74, 1.95) | -0.53 (-3.66, 2.60) | -0.63 (-3.84, 2.57) | 0.87 (-1.53, 3.27) | -1.81 (-4.50, 0.89) | -0.59 (-3.45, 2.27) | -0.64 (-2.60, 1.33) | -0.18 (-3.71, 3.35) |
| RD 40th - Min | -1.21 (-6.05, 3.62) | 0.06 (-2.96, 3.07) | -1.48 (-5.02, 2.06) | -0.56 (-3.88, 2.75) | -0.67 (-4.07, 2.73) | 0.92 (-1.62, 3.46) | -1.92 (-4.77, 0.94) | -0.62 (-3.66, 2.41) | -0.67 (-2.76, 1.41) | -0.19 (-3.93, 3.56) |
| RD 50th - Min | -1.32 (-6.57, 3.93) | 0.06 (-3.21, 3.33) | -1.61 (-5.46, 2.24) | -0.61 (-4.22, 2.99) | -0.73 (-4.43, 2.97) | 1.00 (-1.76, 3.76) | -2.08 (-5.18, 1.02) | -0.68 (-3.98, 2.62) | -0.73 (-2.99, 1.53) | -0.20 (-4.27, 3.86) |
| RD 60th - Min | -1.43 (-7.14, 4.27) | 0.07 (-3.49, 3.62) | -1.75 (-5.93, 2.44) | -0.66 (-4.58, 3.25) | -0.79 (-4.81, 3.22) | 1.09 (-1.91, 4.09) | -2.26 (-5.63, 1.11) | -0.74 (-4.32, 2.85) | -0.80 (-3.25, 1.66) | -0.22 (-4.64, 4.20) |
| RD 70th - Min | -1.54 (-7.66, 4.58) | 0.07 (-3.75, 3.89) | -1.87 (-6.36, 2.61) | -0.71 (-4.91, 3.49) | -0.85 (-5.16, 3.46) | 1.17 (-2.05, 4.39) | -2.43 (-6.04, 1.19) | -0.79 (-4.63, 3.05) | -0.85 (-3.49, 1.78) | -0.24 (-4.98, 4.50) |
| RD 80th - Min | -1.63 (-8.12, 4.86) | 0.08 (-3.97, 4.12) | -1.99 (-6.75, 2.77) | -0.76 (-5.21, 3.70) | -0.90 (-5.47, 3.67) | 1.24 (-2.18, 4.65) | -2.57 (-6.41, 1.26) | -0.84 (-4.91, 3.24) | -0.91 (-3.70, 1.89) | -0.25 (-5.28, 4.78) |
| RD 90th - Min | -1.81 (-9.03, 5.41) | 0.08 (-4.42, 4.58) | -2.21 (-7.50, 3.08) | -0.84 (-5.80, 4.11) | -1.00 (-6.09, 4.08) | 1.38 (-2.42, 5.17) | -2.86 (-7.12, 1.40) | -0.93 (-5.46, 3.60) | -1.01 (-4.12, 2.10) | -0.28 (-5.87, 5.31) |
| RD Max - Min | -2.36 (-11.75, 7.04) | 0.11 (-5.75, 5.97) | -2.88 (-9.77, 4.01) | -1.09 (-7.54, 5.36) | -1.31 (-7.92, 5.31) | 1.79 (-3.15, 6.73) | -3.72 (-9.27, 1.82) | -1.21 (-7.11, 4.69) | -1.31 (-5.36, 2.74) | -0.36 (-7.64, 6.91) |

**Table S5.** Risk differences (RDs) and 95% confidence intervals (CIs) comparing each NDVI exposure decile to minimum NDVI exposure for waist circumference as well as metabolic syndrome for the entire study population as well as stratified by effect modifier (sex, ethnicity, income, and age). Effect modifiers are bolded if Cochran's Q heterogeneity test was found to be statistically significant (p-value < 0.05).

| | Entire Population | Male only | Female only | Hispanic only | Non-Hispanic only | Income <$30k only | Income $30-55k only | Income $55k+ only | **Age 65+ yo only** | **Age <65 yo only** |
|---|---|---|---|---|---|---|---|---|---|---|
| **Waist Circumference** | | | | | | | | | | |
| RD 10th - Min | 0.17 (-4.27, 4.62) | **1.89 (-0.68, 4.47)** | **-2.37 (-5.66, 0.92)** | 0.00 (-3.39, 3.40) | 0.14 (-2.80, 3.08) | -0.76 (-3.54, 2.03) | 1.38 (-0.90, 3.67) | -0.04 (-2.65, 2.56) | -0.29 (-2.09, 1.52) | 0.87 (-2.49, 4.23) |
| RD 20th - Min | 0.20 (-4.97, 5.37) | **2.20 (-0.79, 5.20)** | **-2.76 (-6.59, 1.07)** | 0.00 (-3.94, 3.95) | 0.17 (-3.25, 3.58) | -0.88 (-4.12, 2.36) | 1.61 (-1.04, 4.27) | -0.05 (-3.08, 2.98) | -0.33 (-2.43, 1.76) | 1.02 (-2.89, 4.92) |
| RD 30th - Min | 0.22 (-5.47, 5.91) | **2.43 (-0.87, 5.72)** | **-3.04 (-7.25, 1.18)** | 0.00 (-4.34, 4.35) | 0.18 (-3.58, 3.95) | -0.97 (-4.53, 2.60) | 1.77 (-1.15, 4.70) | -0.05 (-3.39, 3.28) | -0.37 (-2.68, 1.94) | 1.12 (-3.18, 5.42) |
| RD 40th - Min | 0.24 (-5.80, 6.27) | **2.57 (-0.92, 6.07)** | **-3.22 (-7.69, 1.25)** | 0.00 (-4.60, 4.61) | 0.19 (-3.80, 4.18) | -1.03 (-4.80, 2.75) | 1.88 (-1.22, 4.98) | -0.06 (-3.59, 3.48) | -0.39 (-2.84, 2.06) | 1.19 (-3.38, 5.75) |
| RD 50th - Min | 0.26 (-6.30, 6.81) | **2.80 (-1.00, 6.59)** | **-3.50 (-8.36, 1.36)** | 0.00 (-5.00, 5.01) | 0.21 (-4.13, 4.55) | -1.11 (-5.22, 2.99) | 2.04 (-1.32, 5.41) | -0.06 (-3.91, 3.78) | -0.42 (-3.08, 2.24) | 1.29 (-3.67, 6.24) |
| RD 60th - Min | 0.28 (-6.84, 7.40) | **3.04 (-1.09, 7.16)** | **-3.80 (-9.08, 1.48)** | 0.00 (-5.43, 5.44) | 0.23 (-4.48, 4.94) | -1.21 (-5.67, 3.25) | 2.22 (-1.44, 5.88) | -0.07 (-4.24, 4.11) | -0.46 (-3.35, 2.43) | 1.40 (-3.98, 6.78) |
| RD 70th - Min | 0.30 (-7.35, 7.94) | **3.26 (-1.17, 7.69)** | **-4.08 (-9.74, 1.59)** | 0.00 (-5.83, 5.84) | 0.25 (-4.81, 5.30) | -1.30 (-6.09, 3.49) | 2.38 (-1.54, 6.31) | -0.07 (-4.55, 4.41) | -0.49 (-3.60, 2.61) | 1.50 (-4.28, 7.28) |
| RD 80th - Min | 0.32 (-7.79, 8.42) | **3.46 (-1.24, 8.15)** | **-4.32 (-10.33, 1.68)** | 0.01 (-6.19, 6.20) | 0.26 (-5.10, 5.62) | -1.38 (-6.46, 3.70) | 2.53 (-1.64, 6.69) | -0.08 (-4.83, 4.68) | -0.52 (-3.81, 2.76) | 1.59 (-4.53, 7.72) |
| RD 90th - Min | 0.35 (-8.66, 9.37) | **3.84 (-1.38, 9.06)** | **-4.81 (-11.49, 1.87)** | 0.01 (-6.88, 6.89) | 0.29 (-5.67, 6.25) | -1.53 (-7.18, 4.11) | 2.81 (-1.82, 7.44) | -0.08 (-5.37, 5.20) | -0.58 (-4.24, 3.07) | 1.77 (-5.04, 8.58) |
| RD Max - Min | 0.46 (-11.28, 12.19) | **5.00 (-1.79, 11.80)** | **-6.26 (-14.95, 2.44)** | 0.01 (-8.95, 8.97) | 0.38 (-7.38, 8.14) | -1.99 (-9.34, 5.36) | 3.66 (-2.37, 9.68) | -0.11 (-6.99, 6.77) | -0.76 (-5.52, 4.00) | 2.31 (-6.56, 11.17) |
| **Metabolic Syndrome** | | | | | | | | | | |
| RD 10th - Min | 0.09 (-0.47, 0.66) | 0.10 (-0.25, 0.45) | -0.03 (-0.46, 0.41) | -0.17 (-0.61, 0.26) | 0.25 (-0.14, 0.65) | 0.03 (-0.33, 0.39) | 0.20 (-0.16, 0.55) | -0.08 (-0.40, 0.25) | -0.01 (-0.23, 0.20) | 0.17 (-0.38, 0.71) |
| RD 20th - Min | 0.11 (-0.55, 0.77) | 0.12 (-0.29, 0.53) | -0.03 (-0.54, 0.48) | -0.20 (-0.70, 0.30) | 0.30 (-0.16, 0.76) | 0.04 (-0.38, 0.45) | 0.23 (-0.19, 0.64) | -0.09 (-0.47, 0.29) | -0.01 (-0.26, 0.24) | 0.19 (-0.44, 0.83) |
| RD 30th - Min | 0.12 (-0.60, 0.84) | 0.13 (-0.32, 0.58) | -0.03 (-0.59, 0.53) | -0.22 (-0.77, 0.33) | 0.33 (-0.18, 0.83) | 0.04 (-0.42, 0.50) | 0.25 (-0.21, 0.71) | -0.10 (-0.52, 0.31) | -0.02 (-0.29, 0.26) | 0.21 (-0.48, 0.91) |
| RD 40th - Min | 0.13 (-0.64, 0.89) | 0.14 (-0.34, 0.62) | -0.03 (-0.63, 0.56) | -0.24 (-0.82, 0.35) | 0.35 (-0.19, 0.88) | 0.04 (-0.45, 0.53) | 0.27 (-0.22, 0.75) | -0.11 (-0.55, 0.33) | -0.02 (-0.31, 0.28) | 0.23 (-0.51, 0.96) |
| RD 50th - Min | 0.14 (-0.69, 0.97) | 0.15 (-0.37, 0.67) | -0.04 (-0.69, 0.61) | -0.26 (-0.89, 0.38) | 0.38 (-0.21, 0.96) | 0.05 (-0.48, 0.58) | 0.29 (-0.24, 0.81) | -0.12 (-0.59, 0.36) | -0.02 (-0.34, 0.30) | 0.25 (-0.56, 1.05) |
| RD 60th - Min | 0.15 (-0.75, 1.06) | 0.16 (-0.41, 0.73) | -0.04 (-0.74, 0.66) | -0.28 (-0.97, 0.41) | 0.41 (-0.23, 1.04) | 0.05 (-0.53, 0.63) | 0.31 (-0.26, 0.88) | -0.13 (-0.64, 0.39) | -0.02 (-0.37, 0.33) | 0.27 (-0.60, 1.14) |
| RD 70th - Min | 0.16 (-0.81, 1.13) | 0.17 (-0.44, 0.78) | -0.04 (-0.80, 0.71) | -0.30 (-1.04, 0.44) | 0.44 (-0.24, 1.12) | 0.05 (-0.56, 0.67) | 0.34 (-0.28, 0.95) | -0.13 (-0.69, 0.42) | -0.02 (-0.39, 0.35) | 0.29 (-0.65, 1.22) |
| RD 80th - Min | 0.17 (-0.86, 1.20) | 0.18 (-0.46, 0.83) | -0.05 (-0.85, 0.75) | -0.32 (-1.10, 0.47) | 0.46 (-0.26, 1.19) | 0.06 (-0.60, 0.71) | 0.36 (-0.29, 1.01) | -0.14 (-0.73, 0.45) | -0.02 (-0.42, 0.37) | 0.30 (-0.69, 1.29) |
| RD 90th - Min | 0.19 (-0.95, 1.34) | 0.20 (-0.51, 0.92) | -0.05 (-0.94, 0.84) | -0.35 (-1.23, 0.52) | 0.52 (-0.29, 1.32) | 0.06 (-0.67, 0.79) | 0.40 (-0.33, 1.12) | -0.16 (-0.82, 0.50) | -0.02 (-0.46, 0.41) | 0.34 (-0.77, 1.44) |
| RD Max - Min | 0.25 (-1.24, 1.74) | 0.26 (-0.67, 1.20) | -0.07 (-1.23, 1.09) | -0.46 (-1.60, 0.68) | 0.67 (-0.37, 1.72) | 0.08 (-0.87, 1.03) | 0.52 (-0.42, 1.45) | -0.21 (-1.06, 0.65) | -0.03 (-0.60, 0.54) | 0.44 (-1.00, 1.87) |

**Table S6.** Risk differences (RDs) and 95% confidence intervals (CIs) comparing each NDVI exposure decile to minimum NDVI exposure for all outcomes for the entire study population using the super learner ensemble.

| | LDL Cholesterol | Total Cholesterol | Triglycerides | HDL Cholesterol | Glucose | Hemoglobin AIC | Systolic Blood Pressure | Diastolic Blood Pressure | Waist Circumference | Metabolic Syndrome |
|---|---|---|---|---|---|---|---|---|---|---|
| RD 10th - Min | 2.62 (-15.40, 20.64) | -1.40 (-23.78, 20.99) | 10.65 (-7.44, 28.75) | -3.72 (-12.79, 5.35) | -3.50 (-17.09, 10.08) | -0.04 (-0.51, 0.44) | -1.98 (-13.83, 9.88) | -0.58 (-8.09, 6.93) | 1.10 (-17.54, 19.74) | 0.00 (-0.06, 0.06) |
| RD 20th - Min | 6.92 (-10.37, 24.21) | 5.51 (-15.13, 26.16) | 11.97 (-6.60, 30.55) | -2.99 (-13.04, 7.06) | -4.02 (-14.25, 6.21) | -0.05 (-0.44, 0.33) | -2.06 (-13.07, 8.95) | -0.42 (-8.24, 7.41) | 1.13 (-13.88, 16.14) | 0.01 (-0.07, 0.08) |
| RD 30th - Min | 6.05 (-10.02, 22.11) | 3.99 (-16.22, 24.20) | 14.3 (-10.20, 38.80) | -3.72 (-13.72, 6.28) | -4.09 (-17.55, 9.37) | -0.08 (-0.56, 0.40) | -2.28 (-13.79, 9.23) | -0.76 (-8.82, 7.30) | 0.90 (-13.95, 15.76) | 0.02 (-0.09, 0.12) |
| RD 40th - Min | 4.86 (-10.39, 20.10) | 2.91 (-16.36, 22.18) | 15.35 (-3.60, 34.30) | -3.86 (-13.25, 5.54) | -5.56 (-16.50, 5.39) | -0.20 (-0.57, 0.17) | -2.43 (-13.36, 8.50) | -0.51 (-8.32, 7.29) | 1.07 (-14.31, 16.45) | -0.01 (-0.08, 0.06) |
| RD 50th - Min | 5.49 (-11.09, 22.07) | 4.18 (-14.76, 23.11) | 17.54 (-6.17, 41.25) | -3.86 (-13.19, 5.48) | -4.43 (-20.05, 11.20) | -0.11 (-0.52, 0.31) | -2.48 (-13.33, 8.37) | -0.59 (-8.29, 7.12) | 1.32 (-13.19, 15.84) | 0.00 (-0.09, 0.09) |
| RD 60th - Min | 5.46 (-10.20, 21.13) | 4.64 (-13.89, 23.16) | 18.74 (-3.02, 40.50) | -3.77 (-13.95, 6.41) | -5.73 (-15.33, 3.87) | -0.12 (-0.48, 0.23) | -2.60 (-13.34, 8.13) | -0.80 (-8.37, 6.78) | 1.29 (-12.83, 15.41) | -0.01 (-0.08, 0.07) |
| RD 70th - Min | 7.33 (-9.50, 24.17) | 5.87 (-14.00, 25.74) | 20.16 (-0.97, 41.28) | -4.09 (-12.88, 4.71) | -6.20 (-15.85, 3.46) | -0.15 (-0.50, 0.19) | -2.43 (-14.16, 9.29) | -0.41 (-7.84, 7.03) | 1.34 (-13.09, 15.77) | 0.02 (-0.09, 0.13) |
| RD 80th - Min | 8.24 (-9.31, 25.79) | 6.67 (-13.12, 26.46) | 21.2 (-6.01, 48.41) | -3.91 (-13.99, 6.17) | -6.12 (-16.60, 4.36) | -0.12 (-0.51, 0.27) | -2.58 (-14.13, 8.97) | -0.62 (-8.53, 7.29) | 1.29 (-13.26, 15.83) | -0.01 (-0.09, 0.08) |
| RD 90th - Min | 8.24 (-9.31, 25.79) | 6.67 (-13.12, 26.46) | 21.2 (-6.01, 48.41) | -3.91 (-13.99, 6.17) | -6.12 (-16.60, 4.36) | -0.12 (-0.51, 0.27) | -2.58 (-14.13, 8.97) | -0.62 (-8.53, 7.29) | 1.29 (-13.26, 15.83) | -0.01 (-0.09, 0.08) |
| RD Max - Min | 12.26 (-11.99, 36.51) | 9.58 (-13.33, 32.49) | 29.7 (9.00, 50.40) | -2.98 (-12.28, 6.31) | -9.84 (-19.40, -0.27) | -0.20 (-0.59, 0.19) | -1.98 (-14.35, 10.39) | -0.48 (-8.15, 7.19) | 1.06 (-13.43, 15.55) | 0.00 (-0.10, 0.10) |

**1. Expansion of the dataset**

- Keep original dataset (n=558): Block 1
- Create 2 additional copies (total n= 1,674): Blocks 2 & 3

**2. Outcome modeling**

- New dataset used to fit regression model for mean outcome Y given treatment A and confounders L

**3. Prediction**

- Predict outcome values for Blocks 2 and 3 from regression estimates

**4. Standardization by averaging**

- Calculate average of predicted outcomes in Block 2 & in Block 3
- Calculate Risk Difference
- Bootstrapping to obtain 95% confidence interval

Block 1: Original dataset

| ID | A | Y | L |
|---|---|---|---|
| 1 | 1 | 90 | α |
| 2 | 0 | 110 | β |
| ... | ... | ... | ... |
| 558 | 1 | 100 | Δ |

Block 2: A=0

| ID | A | Y | L |
|---|---|---|---|
| 1 | 0 | ___ | α |
| 2 | 0 | ___ | β |
| ... | ... | ... | ... |
| 558 | 0 | ___ | Δ |

Block 2: Average of Predicted Y's

Block 3: A=1

| ID | A | Y | L |
|---|---|---|---|
| 1 | 1 | ___ | α |
| 2 | 1 | ___ | β |
| ... | ... | ... | ... |
| 558 | 1 | ___ | Δ |

Block 3: Average of Predicted Y's

Risk Difference: Average $Y_{Block\ 3}$ – Average $Y_{Block\ 2}$

**Regression model: Y = A + L**

where Y = Blood Glucose; A = NDVI;

L = Sex, Income, Race, Ethnicity, Age, Education

References:
Hernan & Robins, 2020;
Taubman et al., 2009

**Figure S1.** Steps to apply g-computation using this study as the example (Hernán & Robins, 2020; Taubman et al., 2009).

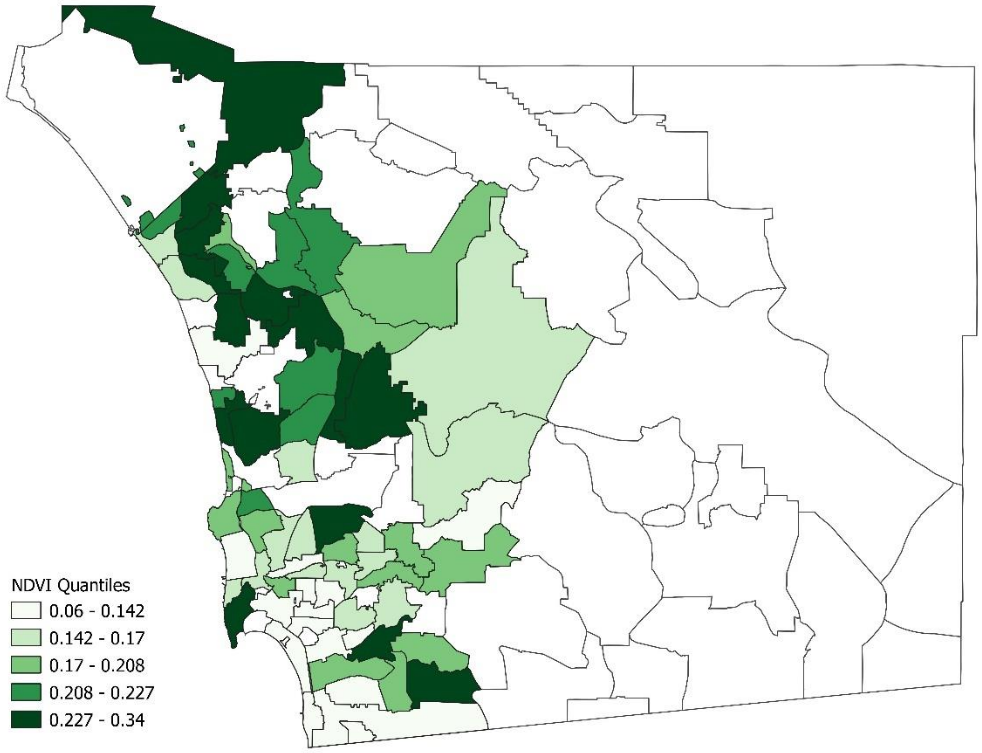


**Figure S2a.** Map depicting the quantiles of NDVI exposure for San Diego County at the ZIP code level.

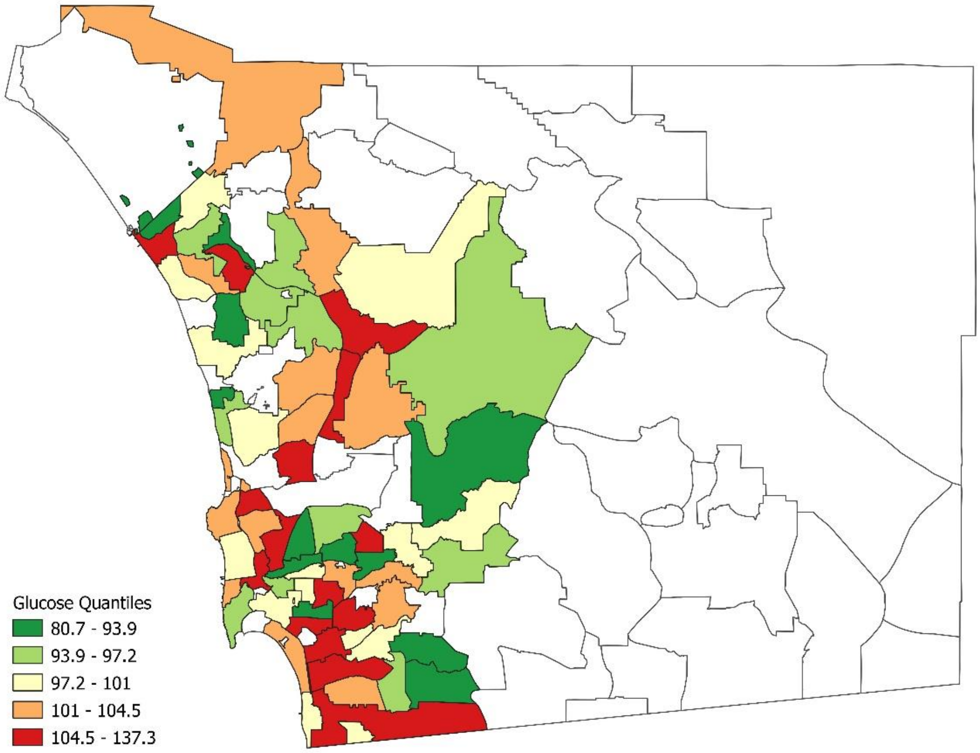


**Figure S2b.** Map depicting the quantiles of glucose (mg/dL) for San Diego County at the ZIP code level.

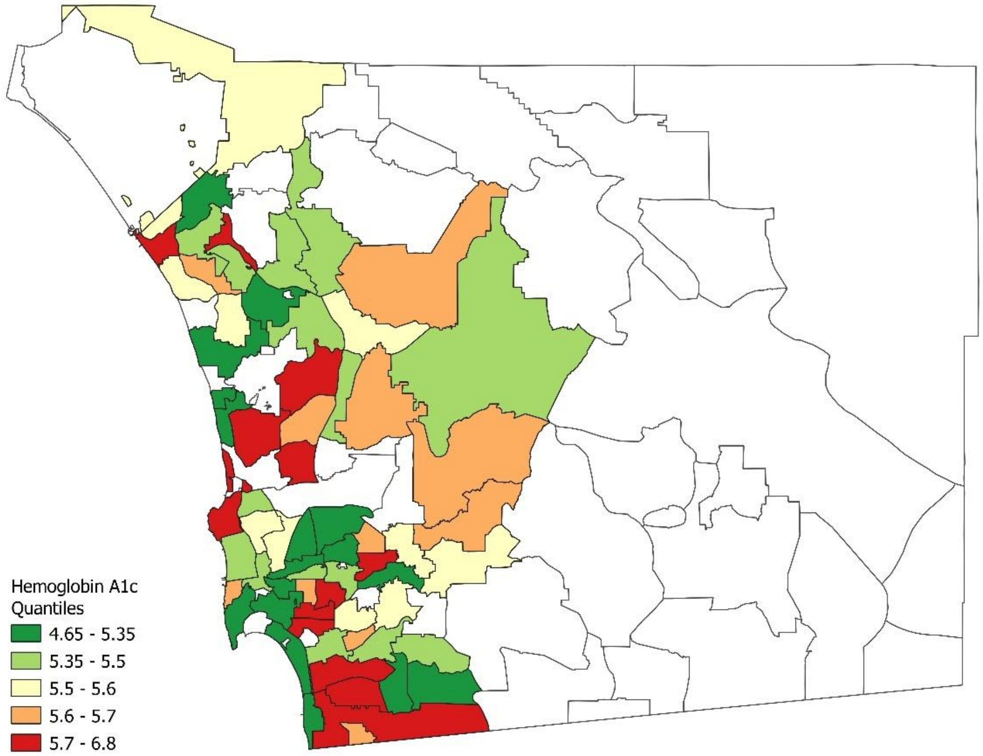


**Figure S2c.** Map depicting the quantiles of hemoglobin A1c % for San Diego County at the ZIP code level.

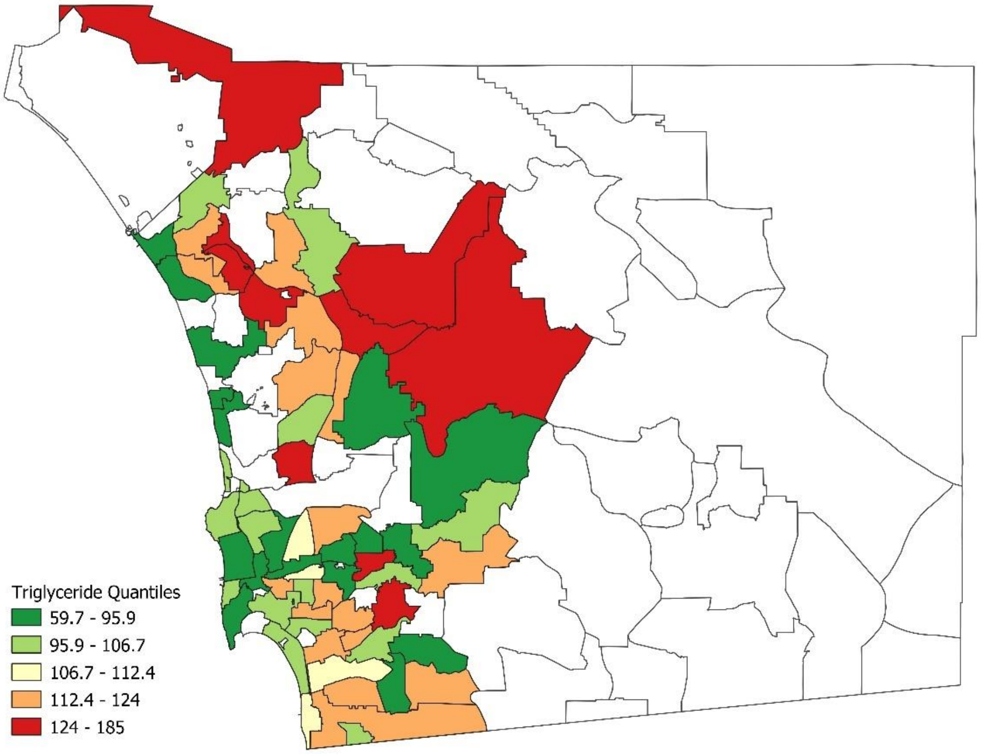


**Figure S2d.** Map depicting the quantiles of triglyceride concentrations (mg/dL) for San Diego County at the ZIP code level.

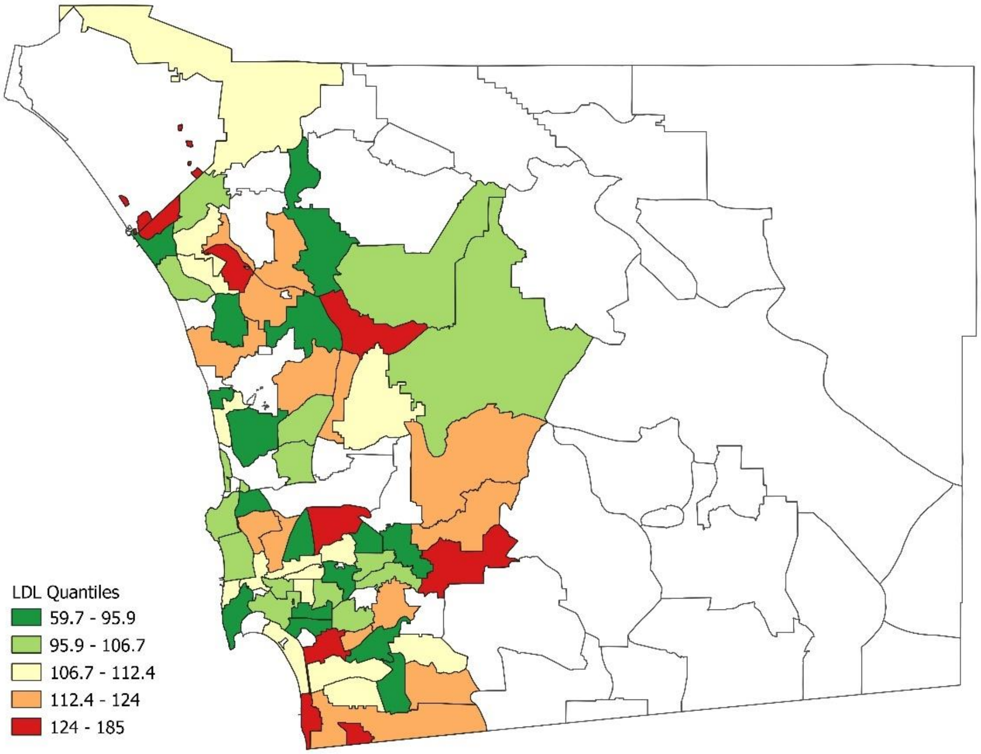


**Figure S2e.** Map depicting the quantiles of LDL cholesterol concentrations (mg/dL) for San Diego County at the ZIP code level.

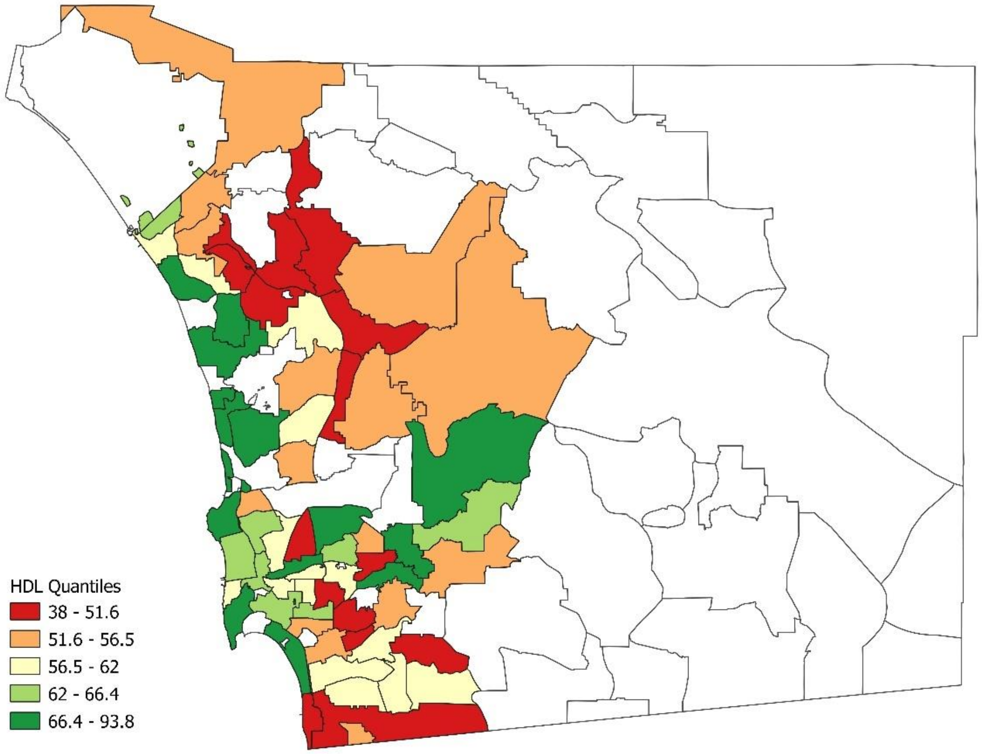


**Figure S2f.** Map depicting the quantiles of HDL cholesterol concentrations (mg/dL) for San Diego County at the ZIP code level.

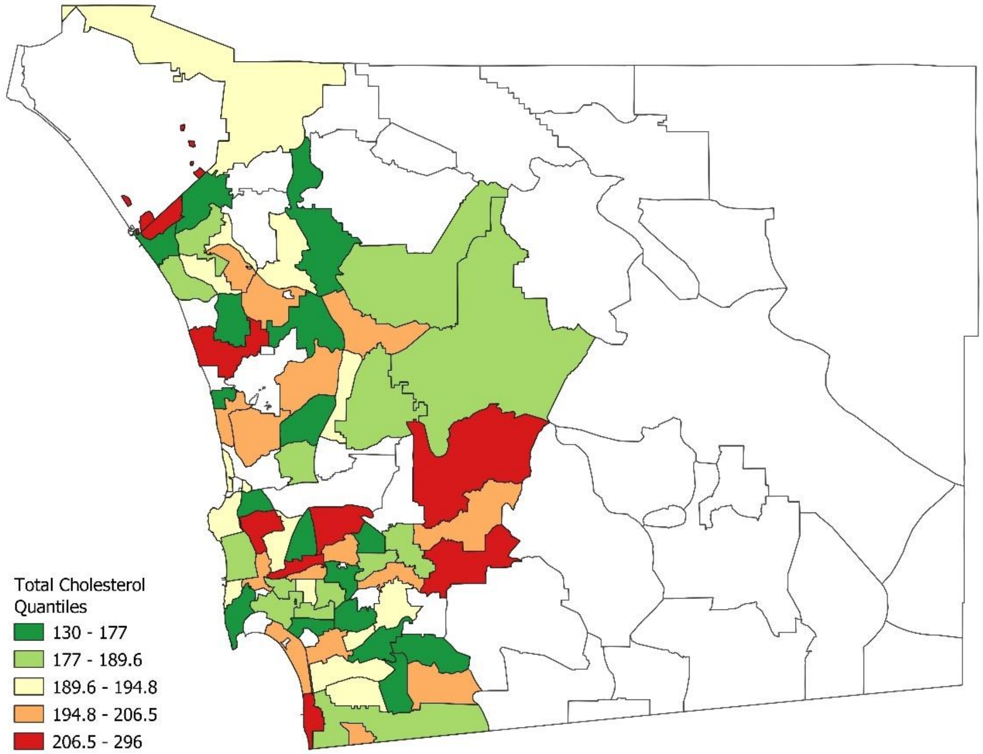


**Figure S2g.** Map depicting the quantiles of total cholesterol concentrations (mg/dL) for San Diego County at the ZIP code level.

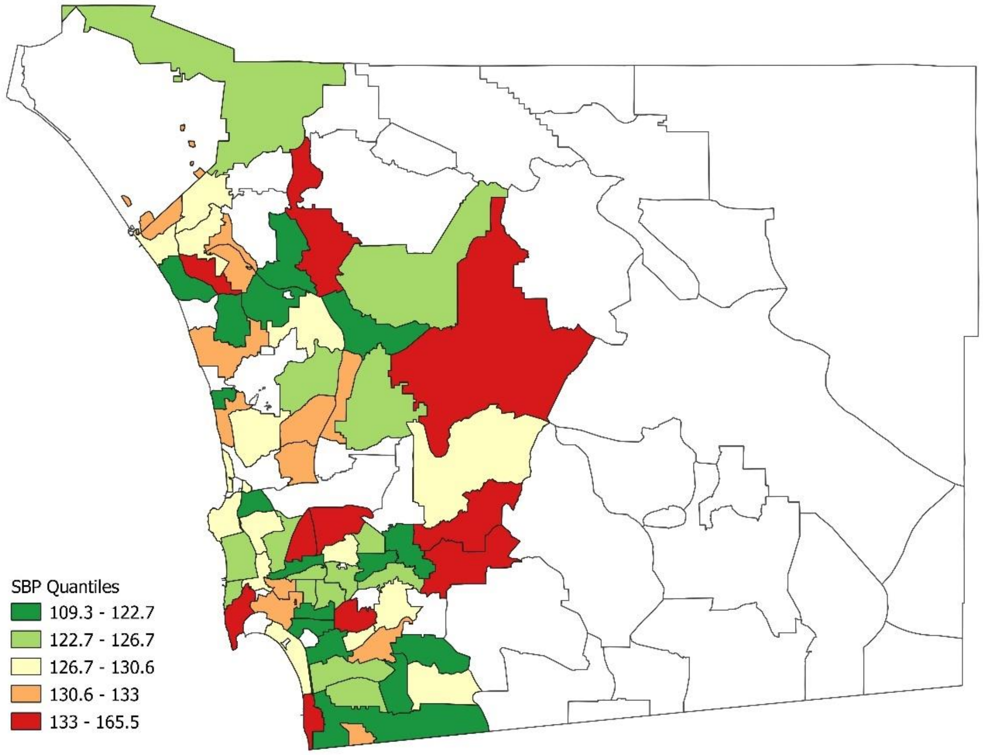


**Figure S2h.** Map depicting the quantiles of systolic blood pressure (mmHg) for San Diego County at the ZIP code level.

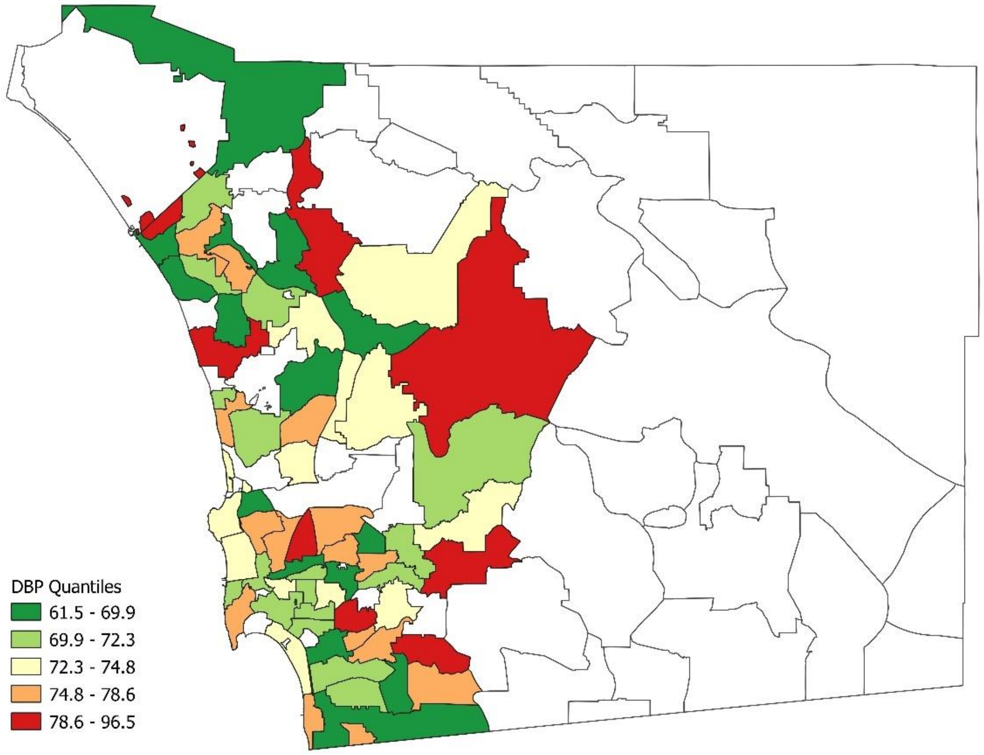


**Figure S2i.** Map depicting the quantiles of diastolic blood pressure (mmHg) for San Diego County at the ZIP code level.

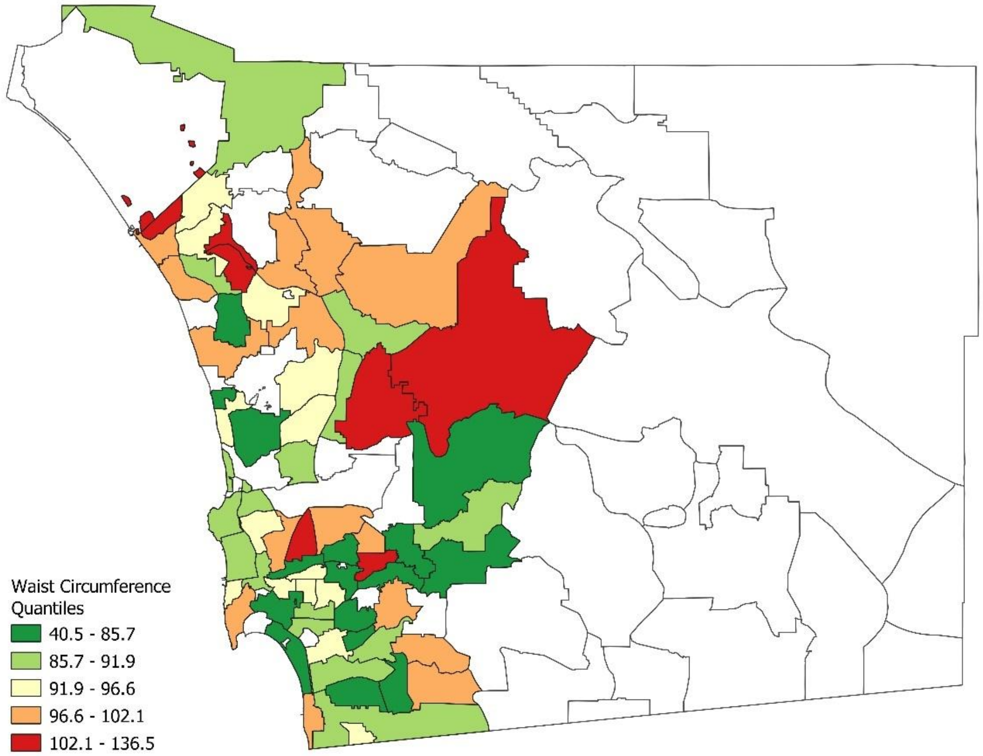


**Figure S2j.** Map depicting the quantiles of waist circumference (cm) for San Diego County at the ZIP code level.

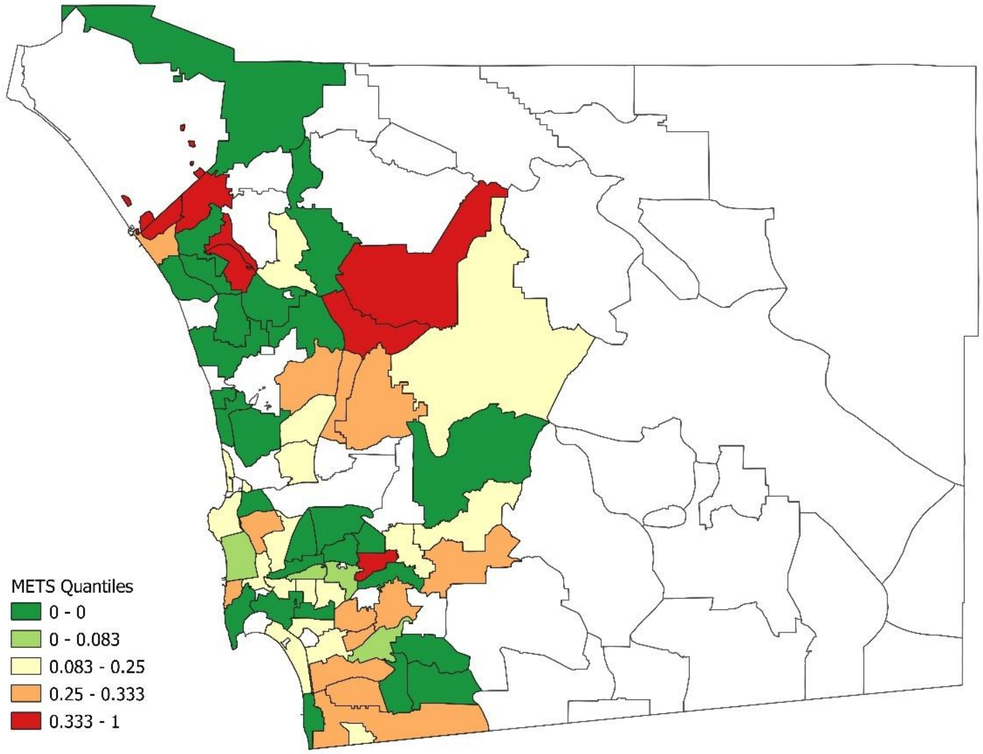


**Figure S2k.** Map depicting the quantiles of metabolic syndrome (as a proportion) for San Diego County at the ZIP code level.

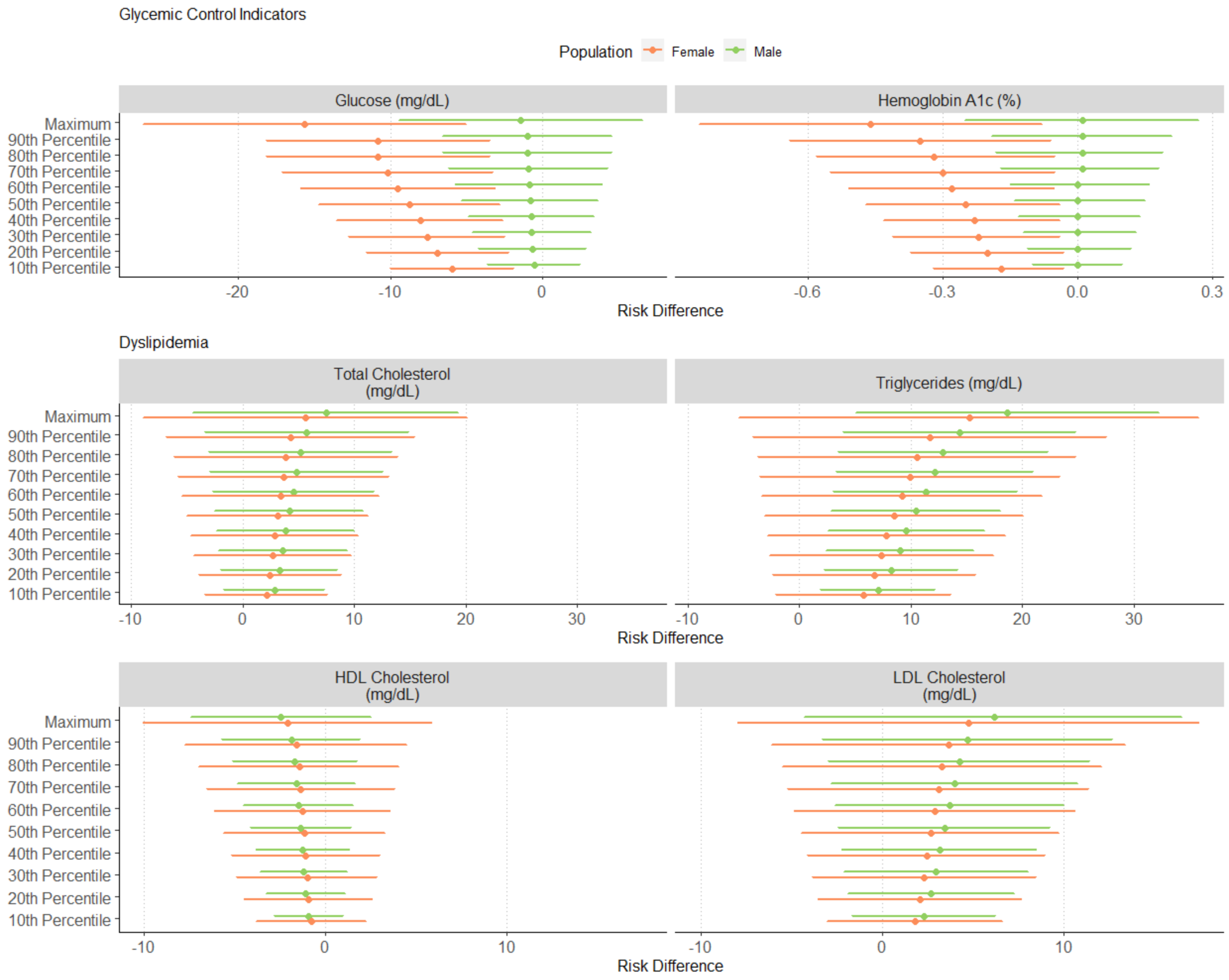
Glycemic Control Indicators
Population
Female
Male
Glucose (mg/dL)
Hemoglobin A1c (%)
Maximum
90th Percentile
80th Percentile
70th Percentile
60th Percentile
50th Percentile
40th Percentile
30th Percentile
20th Percentile
10th Percentile
-20
-10
0
-0.6
-0.3
0.0
0.3
Risk Difference
Dyslipidemia
Total Cholesterol (mg/dL)
Triglycerides (mg/dL)
-10
0
10
20
30
Risk Difference
HDL Cholesterol (mg/dL)
LDL Cholesterol (mg/dL)
-10
0
10
Risk Difference

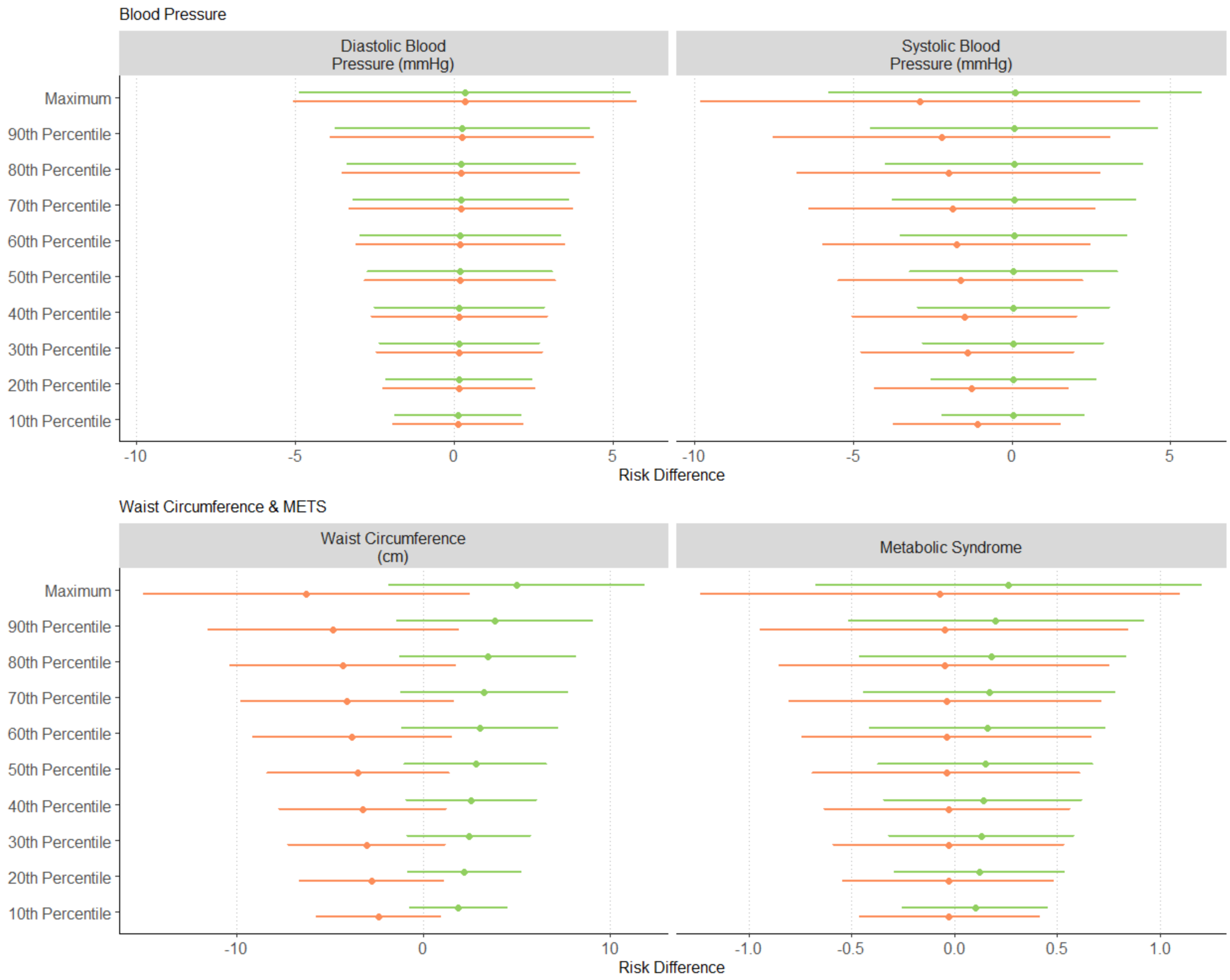


**Figure S3.** Risk differences (RDs) of average biomarker changes with simulated changes to NDVI exposure, stratified by sex. RDs are shown as points and 95% confidence intervals (CIs) as lines on the x-axis and simulated NDVI exposure on the y-axis as deciles compared to minimum NDVI exposure for the glycemic control indicators (top row from left to right: fasting glucose levels and hemoglobin A1c), dyslipidemia (2nd row from left to right: total cholesterol and triglycerides; 3rd row from left to right: HDL cholesterol and LDL cholesterol) blood pressure (4th row from left to right: diastolic blood pressure and systolic blood pressure), and waist circumference and METS (bottom row from left to right) outcomes. Effect modification by sex depicted, where males are shown in green and females are shown in orange.

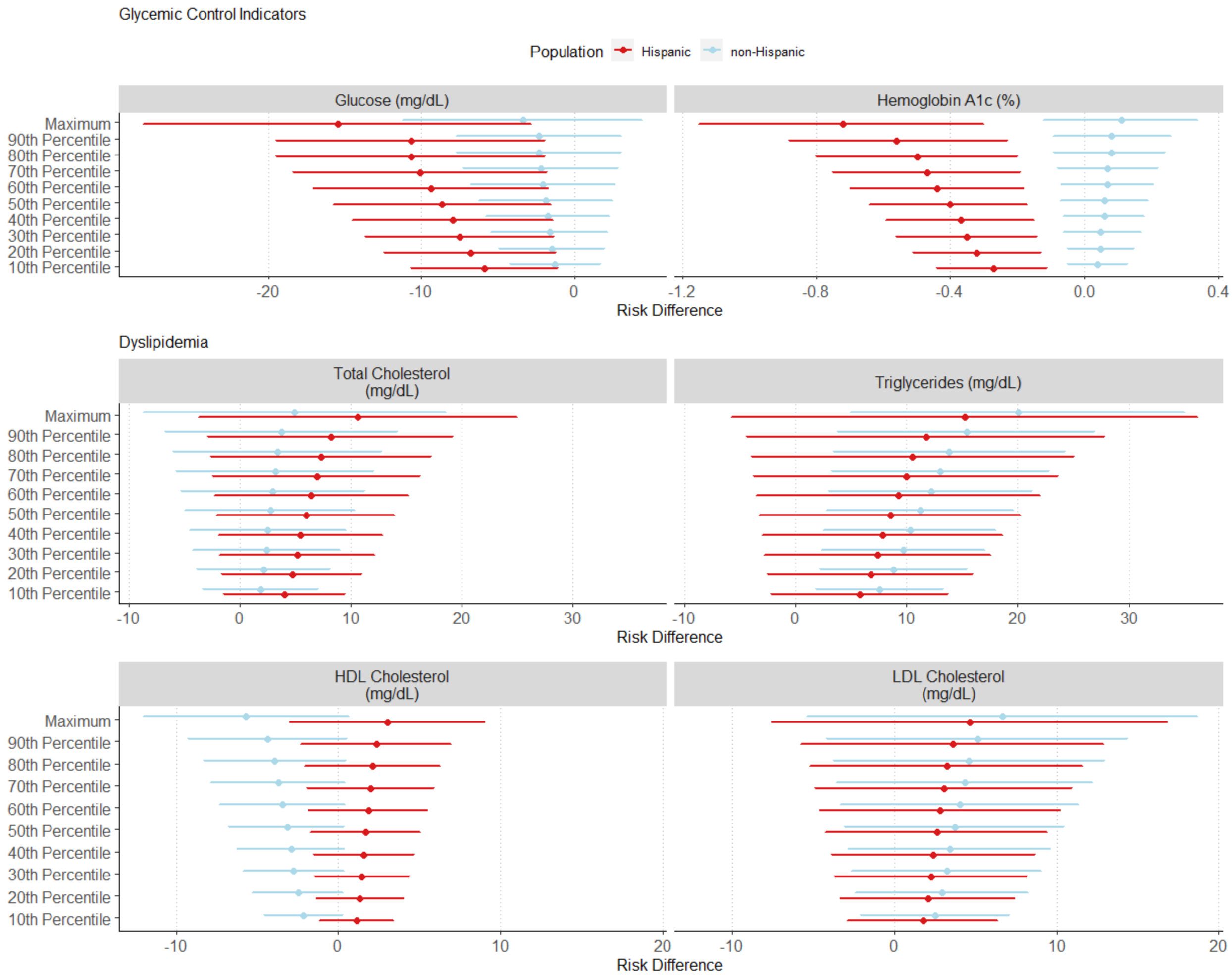

Glycemic Control Indicators
Population
Hispanic
non-Hispanic
Glucose (mg/dL)
Hemoglobin A1c (%)
Maximum
90th Percentile
80th Percentile
70th Percentile
60th Percentile
50th Percentile
40th Percentile
30th Percentile
20th Percentile
10th Percentile
-20
-10
0
-1.2
-0.8
-0.4
0.0
0.4
Risk Difference
Dyslipidemia
Total Cholesterol (mg/dL)
Triglycerides (mg/dL)
-10
0
10
20
30
Risk Difference
HDL Cholesterol (mg/dL)
LDL Cholesterol (mg/dL)
-10
0
10
20
Risk Difference

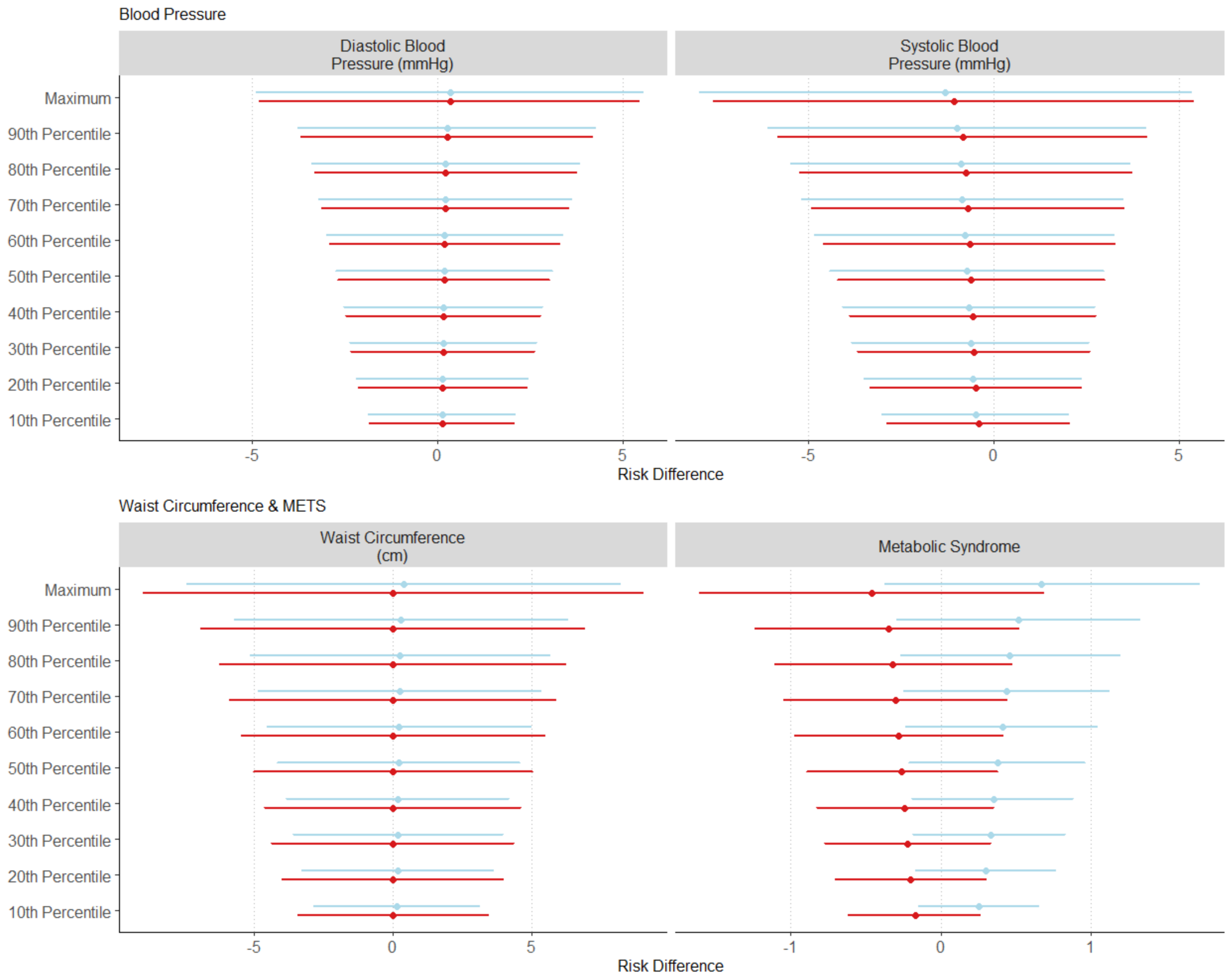


**Figure S4.** Risk differences (RDs) of average biomarker changes with simulated changes to NDVI exposure, stratified by ethnicity. RDs are shown as points and 95% confidence intervals (CIs) as lines on the x-axis and simulated NDVI exposure on the y-axis as deciles compared to minimum NDVI exposure for the glycemic control indicators (top row from left to right: fasting glucose levels and hemoglobin A1c), dyslipidemia (2nd row from left to right: total cholesterol and triglycerides; 3rd row from left to right: HDL cholesterol and LDL cholesterol) blood pressure (4th row from left to right: diastolic blood pressure and systolic blood pressure), and waist circumference and METS (bottom row from left to right) outcomes. Effect modification by ethnicity depicted, where Hispanic participants are shown in red and non-Hispanic participants are shown in blue.

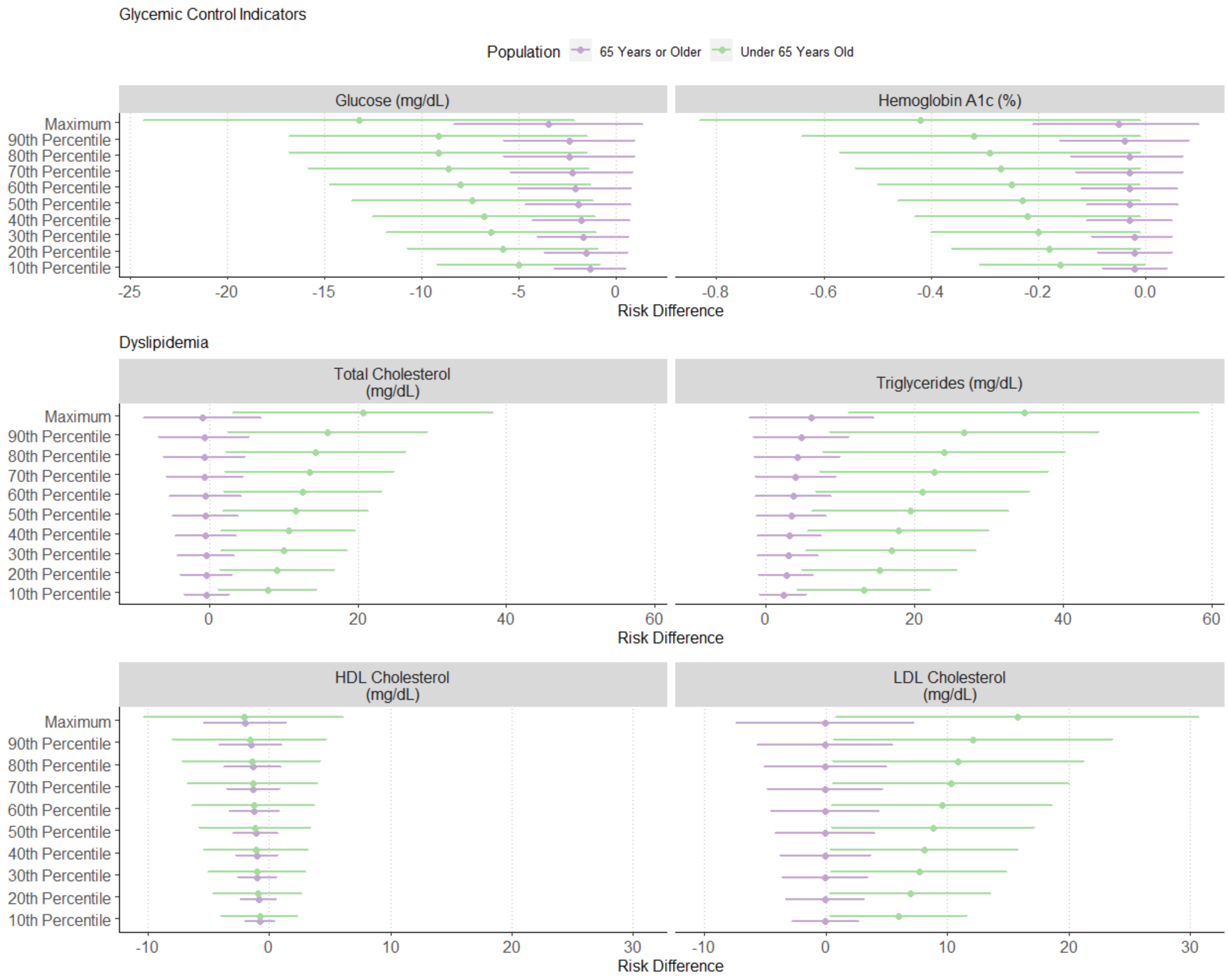
Glycemic Control Indicators
Population
65 Years or Older
Under 65 Years Old
Glucose (mg/dL)
Hemoglobin A1c (%)
Maximum
90th Percentile
80th Percentile
70th Percentile
60th Percentile
50th Percentile
40th Percentile
30th Percentile
20th Percentile
10th Percentile
-25
-20
-15
-10
-5
0
-0.8
-0.6
-0.4
-0.2
0.0
Risk Difference
Dyslipidemia
Total Cholesterol (mg/dL)
Triglycerides (mg/dL)
0
20
40
60
Risk Difference
HDL Cholesterol (mg/dL)
LDL Cholesterol (mg/dL)
-10
0
10
20
30
Risk Difference

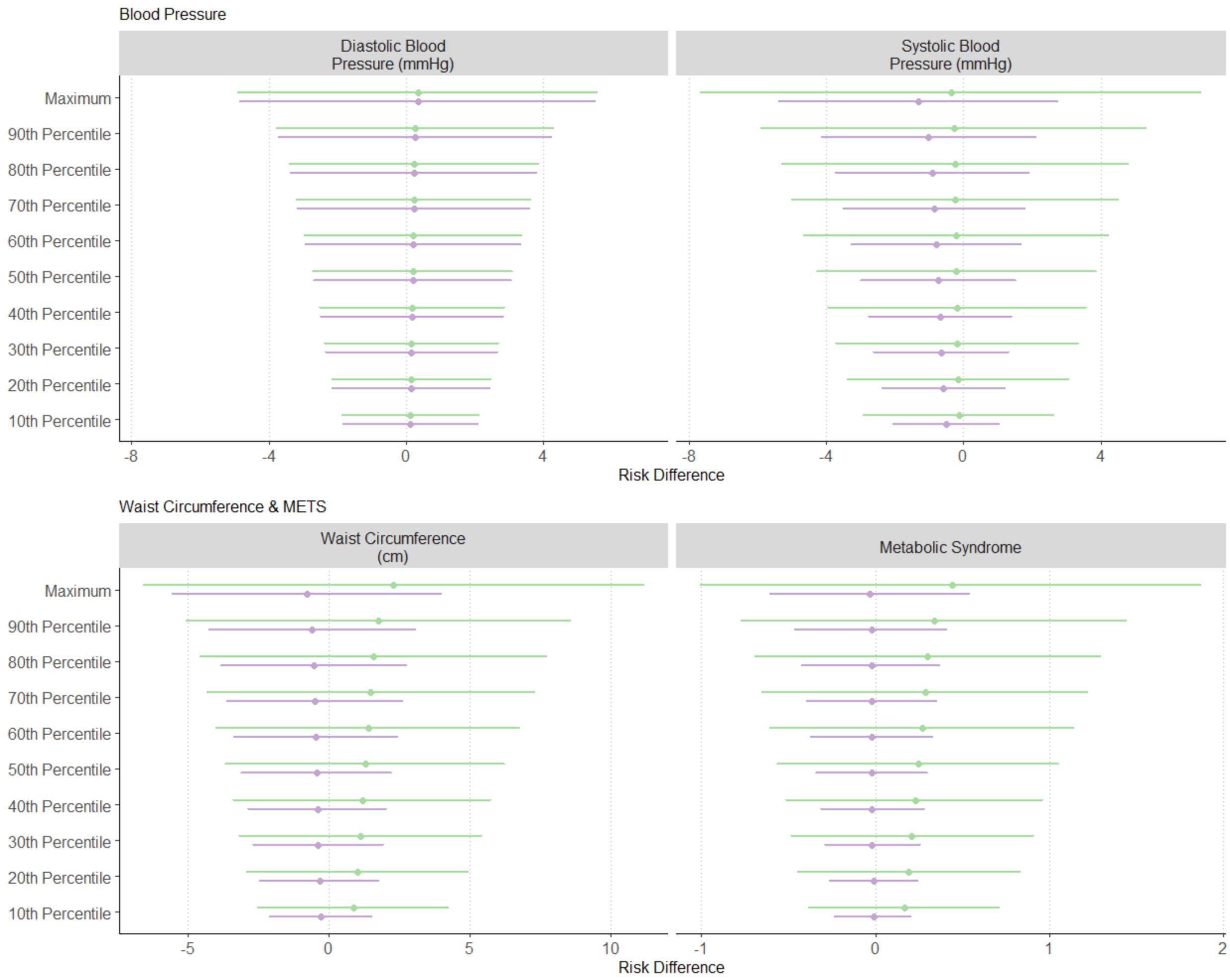


**Figure S5.** Risk differences (RDs) of average biomarker changes with simulated changes to NDVI exposure, stratified by age. RDs are shown as points and 95% confidence intervals (CIs) as lines on the x-axis and simulated NDVI exposure on the y-axis as deciles compared to minimum NDVI exposure for the glycemic control indicators (top row from left to right: fasting glucose levels and hemoglobin A1c), dyslipidemia (2nd row from left to right: total cholesterol and triglycerides; 3rd row from left to right: HDL cholesterol and LDL cholesterol) blood pressure (4th row from left to right: diastolic blood pressure and systolic blood pressure), and waist circumference and METS (bottom row from left to right) outcomes. Effect modification by age depicted, where those 65 years or older are shown in purple and those under 65 years old are shown in green.

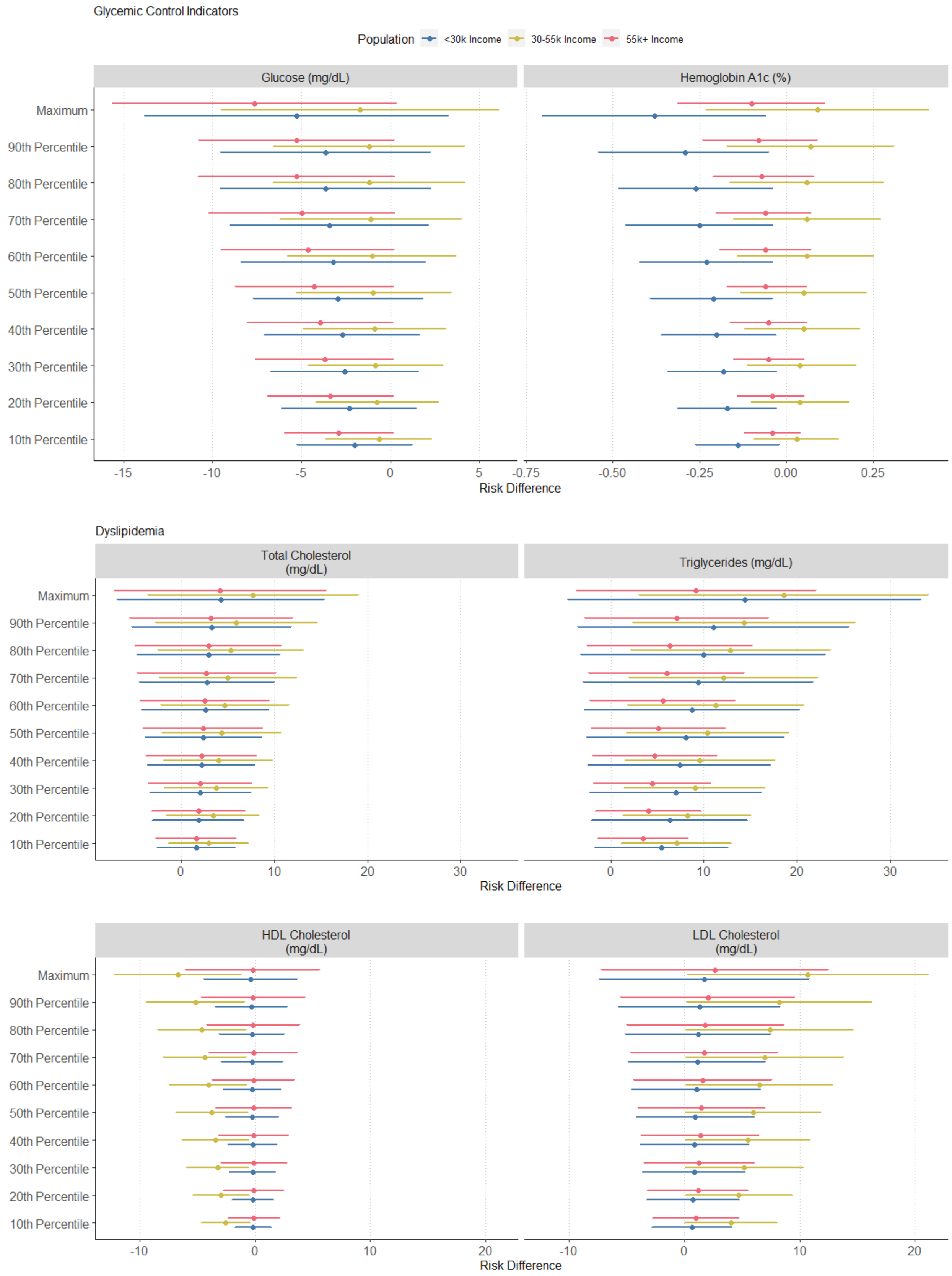
Glycemic Control Indicators
Population
<30k Income
30-55k Income
55k+ Income
Glucose (mg/dL)
Hemoglobin A1c (%)
Maximum
90th Percentile
80th Percentile
70th Percentile
60th Percentile
50th Percentile
40th Percentile
30th Percentile
20th Percentile
10th Percentile
-15
-10
-5
0
5
-0.75
-0.50
-0.25
0.00
0.25
Risk Difference
Dyslipidemia
Total Cholesterol (mg/dL)
Triglycerides (mg/dL)
0
10
20
30
Risk Difference
HDL Cholesterol (mg/dL)
LDL Cholesterol (mg/dL)
-10
0
10
20
Risk Difference

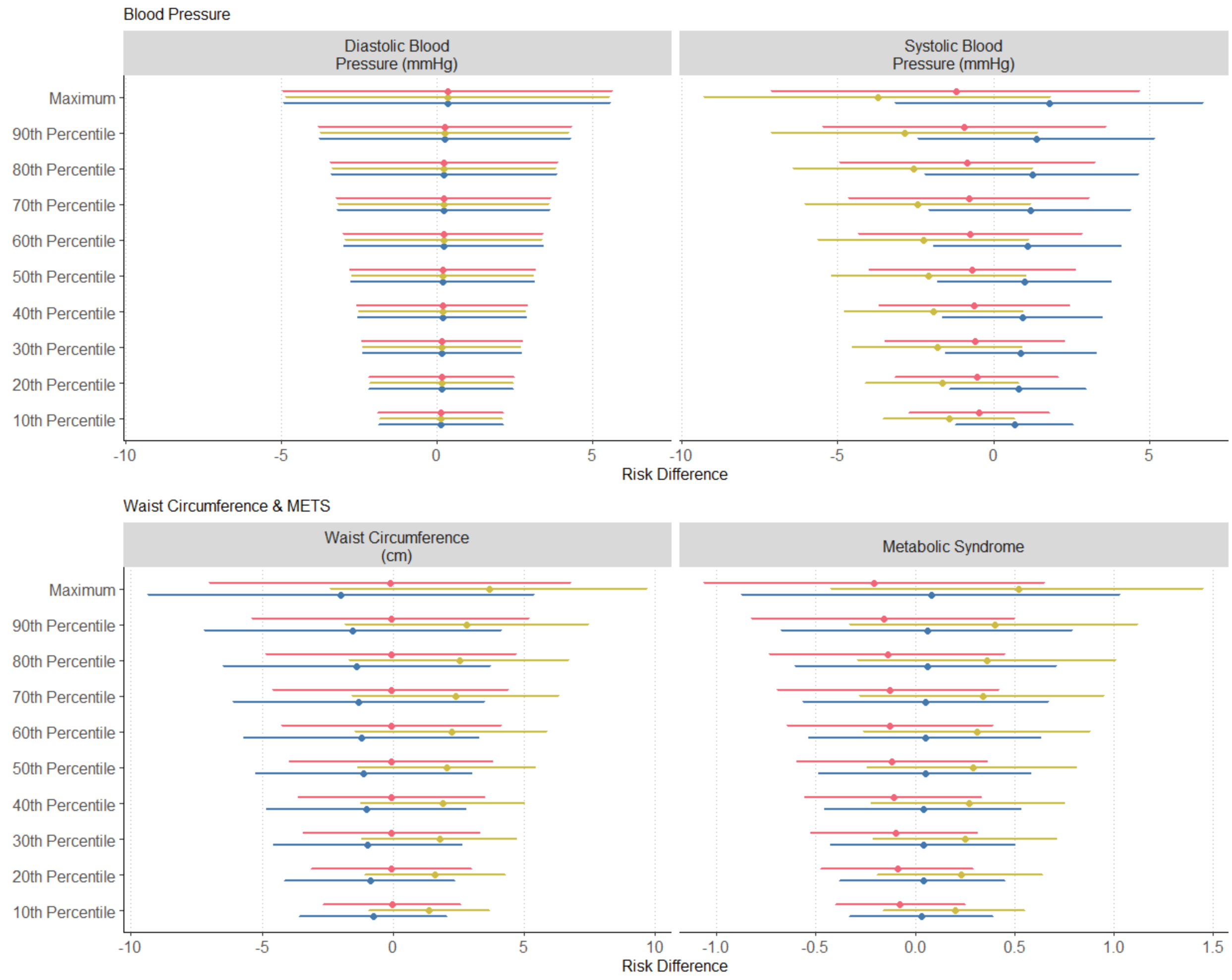


**Figure S6.** Risk differences (RDs) of average biomarker changes with simulated changes to NDVI exposure, stratified by income. RDs are shown as points and 95% confidence intervals (CIs) as lines on the x-axis and simulated NDVI exposure on the y-axis as deciles compared to minimum NDVI exposure for the glycemic control indicators (top row from left to right: fasting glucose levels and hemoglobin A1c), dyslipidemia (2nd row from left to right: total cholesterol and triglycerides; 3rd row from left to right: HDL cholesterol and LDL cholesterol) blood pressure (4th row from left to right: diastolic blood pressure and systolic blood pressure), and waist circumference and METS (bottom row from left to right) outcomes. Effect modification by income depicted, where an annual income under \$30,000 is shown in blue, \$30,000-55,000 is shown in yellow, and \$55,000 or more is shown in red.

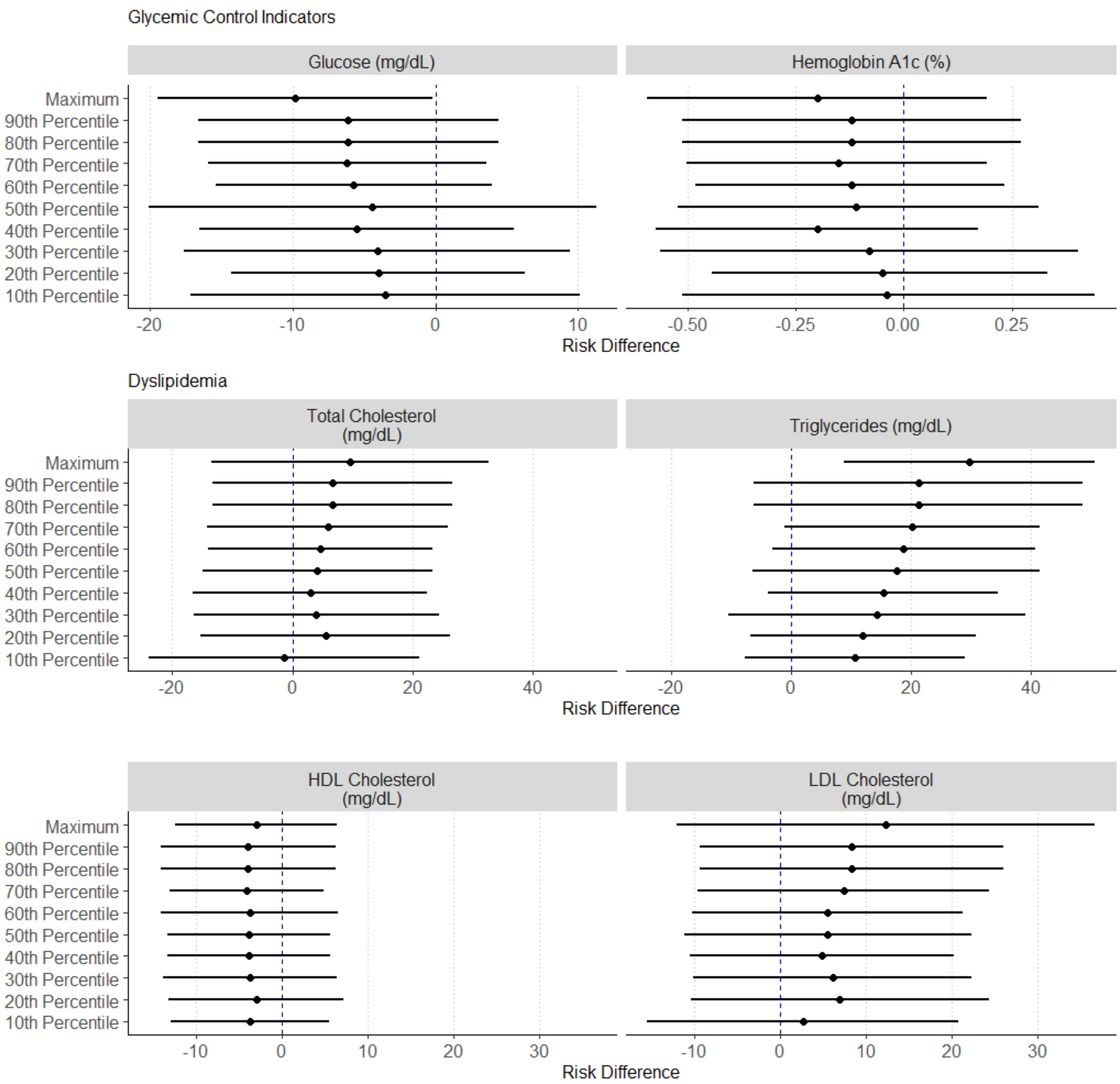

Glycemic Control Indicators
Glucose (mg/dL)
Hemoglobin A1c (%)
Maximum
90th Percentile
80th Percentile
70th Percentile
60th Percentile
50th Percentile
40th Percentile
30th Percentile
20th Percentile
10th Percentile
-20
-10
0
10
-0.50
-0.25
0.00
0.25
Risk Difference
Dyslipidemia
Total Cholesterol (mg/dL)
Triglycerides (mg/dL)
-20
0
20
40
Risk Difference
HDL Cholesterol (mg/dL)
LDL Cholesterol (mg/dL)
-10
0
10
20
30
Risk Difference

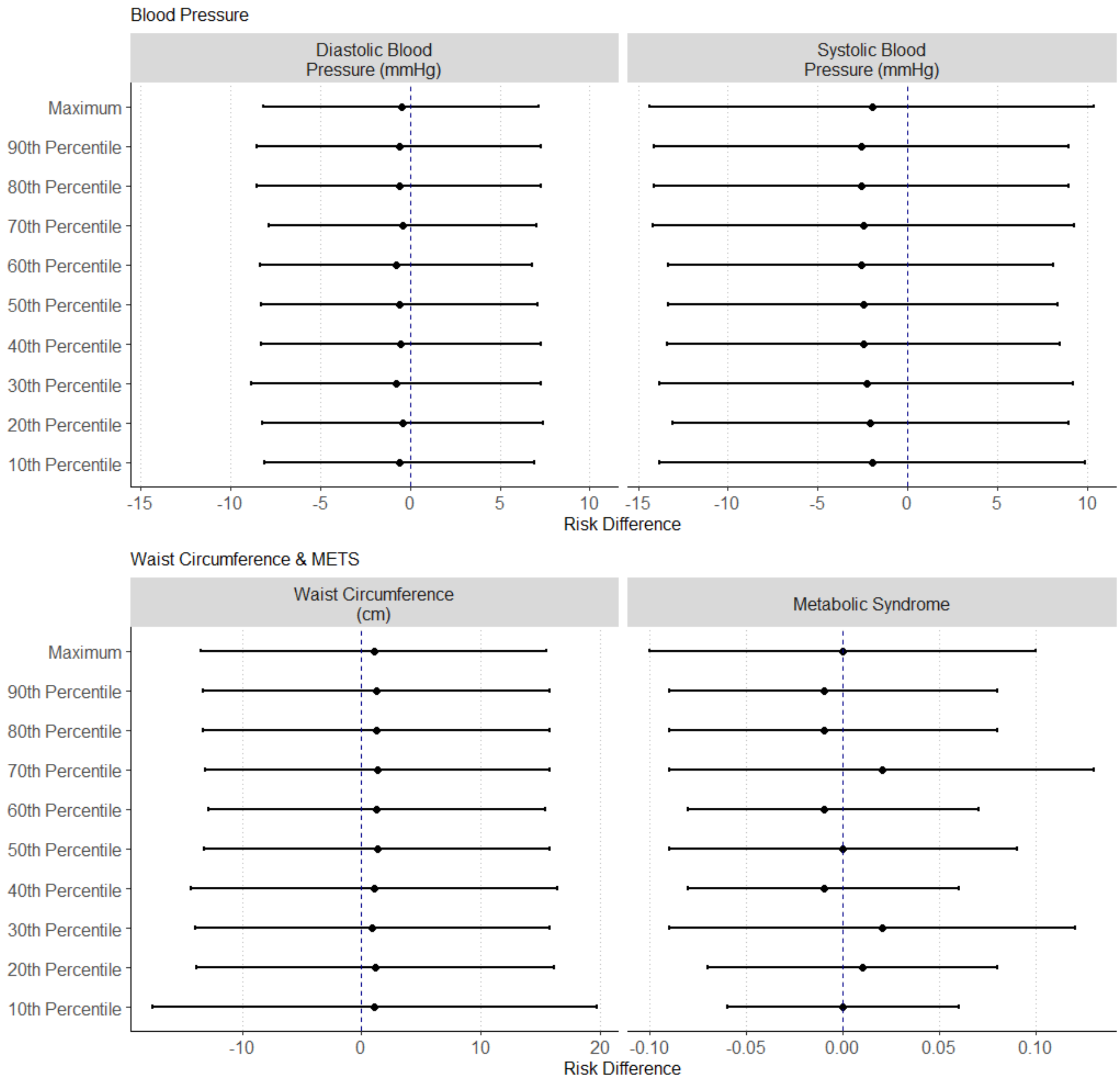


**Figure S7**. Risk differences (RDs) of average biomarker changes with simulated changes to NDVI exposure for the total population using the super learner ensemble. RDs are shown as points and 95% confidence intervals (CIs) as lines on the x-axis and simulated NDVI exposure on the y-axis as deciles compared to minimum NDVI exposure for the glycemic control indicators (top row from left to right: fasting glucose levels and hemoglobin A1c), dyslipidemia (2$^{nd}$ row from left to right: total cholesterol and triglycerides; 3$^{rd}$ row from left to right: HDL cholesterol and LDL cholesterol) blood pressure (4$^{th}$ row from left to right: diastolic blood pressure and systolic blood pressure), and waist circumference and METS (bottom row from left to right) outcomes.
